# CRISPR/Cas9‐induced disruption of *Bodo saltans* paraflagellar rod‐2 gene reveals its importance for cell survival

**DOI:** 10.1111/1462-2920.15918

**Published:** 2022-02-02

**Authors:** Fatma Gomaa, Zhu‐Hong Li, David J. Beaudoin, Heba Alzan, Peter R. Girguis, Roberto Docampo, Virginia P. Edgcomb

**Affiliations:** ^1^ Department of Geology and Geophysics Woods Hole Oceanographic Institution Woods Hole MA 02543 USA; ^2^ Department of Organismic and Evolutionary Biology Harvard University Cambridge MA 02138 USA; ^3^ Department of Cellular Biology and Center for Tropical and Emerging Global Diseases University of Georgia Athens GA USA; ^4^ Department of Veterinary Microbiology and Pathology, College of Veterinary Medicine Washington State University Pullman WA USA; ^5^ Parasitology and Animal Diseases Department, National Research Center Giza Egypt

## Abstract

Developing transfection protocols for marine protists is an emerging field that will allow the functional characterization of protist genes and their roles in organism responses to the environment. We developed a CRISPR/Cas9 editing protocol for *Bodo saltans*, a free‐living kinetoplastid with tolerance to both marine and freshwater conditions and a close non‐parasitic relative of trypanosomatids. Our results show that SaCas9/single‐guide RNA (sgRNA) ribonucleoprotein (RNP) complex‐mediated disruption of the paraflagellar rod 2 gene (*BsPFR2*) was achieved using electroporation‐mediated transfection. The use of CRISPR/Cas9 genome editing can increase the efficiency of targeted homologous recombination when a repair DNA template is provided. Our sequence analysis suggests two mechanisms for repairing double‐strand breaks in *B. saltans* are active; homologous‐directed repair (HDR) utilizing an exogenous DNA template that carries an antibiotic resistance gene and likley non‐homologous end joining (NHEJ). However, HDR was only achieved when a single (vs. multiple) SaCas9 RNP complex was provided. Furthermore, the biallelic knockout of *BsPFR2* was detrimental for the cell, highlighting its essential role for cell survival because it facilitates the movement of food particles into the cytostome. Our Cas9/sgRNA RNP complex protocol provides a new tool for assessing gene functions in *B. saltans* and perhaps similar protists with polycistronic transcription.

## Introduction

Protists are a morphologically and functionally diverse group of eukaryotic microbes, which includes parasites, heterotrophs, autotrophs and mixotrophs. The vast majority are single‐celled and possess diverse and fascinating subcellular features such as: modified mitochondrial DNA, known as the kinetoplast in kinetoplastids; a specialized nucleus in dinoflagellates known as a dinokaryon; and nuclear dualism in many ciliates (Katz, 2001; Shlomai, 2004; Gornik *et al*., 2012). Protists exhibit life cycles that are distinctive for individual groups and sometimes even for individual species (Sherr *et al*., 2007; Adl *et al*., 2019). Free‐living protists play important roles in terrestrial and aquatic ecosystems, contributing to nutrient and biogeochemical cycling (e.g., carbon, nitrogen) (Edgcomb, 2016; Gao *et al*., 2019). Some have evolved to inhabit harsh environments and to contribute to biogeochemical cycles of significant ecological interest (e.g., denitrification, ammonium assimilation) (Gomaa *et al*., 2021). Many species establish a symbiotic relationship with bacteria and archaea as a proxy for metabolic innovations (Douglas, 2014; Husnik *et al*., 2021), while others have the capacity to form multicellular colonies and to display complex cellular organizations that provide an opportunity to study how multicellular organisms may have evolved from protist ancestors (Kirk, 2005; Cavalier‐Smith, 2017). With all these diverse characteristics and evolutionary successes, we know surprisingly little about the genetic repertoire of many protist species. Barriers to this have included a paucity of high‐quality genetic data from many protist groups, as well as a significant fraction of genetic content with unknown function. Successful genetic tools have not been developed for a majority of protist groups, and in the absence of model organisms for major lineages, elucidation of the function of this genetic ‘dark matter’ is difficult, and impedes our understanding of the ecological roles of protists.

Recently, the Gordon and Betty Moore Foundation recognized the importance of developing representative marine protists into model systems and established the Environmental Model Systems project. Gene editing and transformation protocols for several species from major groups of protists were reported from this effort that have extended our knowledge of genome editing methods and challenges for those protists (Faktorová *et al*., 2020).

A successful transient transfection protocol was reported by our group for *Parabodo caudatus* (Metakinetoplastina, Kinetoplastea, Discoba), a free‐living biflagellate protist. This protocol utilized electroporation with square wave pulses and plasmids carrying eukaryotic promoters [Elongation Factor 1 Alpha (EF‐1 alpha) or Ubiquitin C) (UBC)] and an enhanced green fluorescent protein gene (*eGFP*) (Gomaa *et al*., 2017). Because the *Parabodo caudatus* genome is not published, this limited our ability to utilize the same species to move forward to develop stable transfection protocols. Genome sequences reveal genome structure and organization and are necessary to identify likely promoters and to examine how the species regulates transcription, all of which are critical for developing stable transfection methods and precise gene editing protocols. A close relative species to *Parabodo caudatus* with an available genome sequence is *Bodo saltans* (Jackson *et al*., 2008). *Bodo saltans* is an abundant species in both freshwater and marine environments (Opperdoes *et al*., 2016), and it received attention recently in studies focusing on its symbionts. A bacterial endosymbiont of *B. saltans* belongs to the *Alphaproteobacteria* and encodes genes for toxin–antitoxin systems. This symbiont represents a case of evolved mutual dependency; the symbiont protects its host, but also is ensured of its ability to spread throughout the host population (Midha *et al*., 2021). *Bodo saltans* is also known to host one of the largest Mimiviridae viruses (Deeg *et al*., 2018).

The genome of *B. saltans* shares several structural features with trypanosomatid kinetoplastids (Jackson *et al*., 2008; Jackson *et al*., 2016; Butenko *et al*., 2020). Both groups operate polycistronic transcription mechanisms and share significant conserved gene synteny (Jackson *et al*., 2008). Because *trans*‐splicing and polyadenylation are coupled processes for maturating RNA transcripts that contain many loci, deletion of the *trans*‐splicing regulators can cause errors or can prevent mRNA translation entirely. It is therefore challenging to develop stable transfection protocols that integrate vector DNA into the genomes of kinetoplastids, although progress in the genetic manipulation of parasitic kinetoplastids has been reported (Matthews, 2015). One approach for overcoming this problem is to transfect cells with a promoter‐less vector that includes a regulatory intergenic region for polyadenylation. We applied this approach, and successfully established transfection in *B. saltans* using a plasmid that contains a cassette designed to fuse an endogenous elongation factor 1‐alpha (*EF1‐α*) gene with the *eGFP* gene for C‐terminal tagging (Faktorová *et al*., 2020). For this *B. saltans* transfection we placed the tubulin intergenic region downstream of the *eGFP* gene and this was followed by the selectable marker gene neomycin phosphotransferase II (*nptII/neo*), conferring resistance to neomycin. Genotyping analysis of transformants confirmed the presence of the *nptII/neo* gene and the presence of at least part of the plasmid sequence. Plasmid integration into the *B. saltans* genome through homologous recombination remained unconfirmed. This suggests occurrence of off‐target plasmid integration or episomal maintenance. The fact that we did not detect plasmid integration by homologous recombination even with the presence of a DNA template with sufficient flanking sequences suggests that an active homology directed‐repair (HDR) mechanism may be rare in *B. saltans*, as reported in another kinetoplastid species, *Trypanosoma cruzi* (Peng *et al*., 2014).

The CRISPR/Cas9 system for gene editing has been shown to be a rapid and efficient method for inducing double‐stranded breaks (DSBs) in *T. cruzi* when used in conjunction with a repair DNA template with homologous recombination arms (Peng *et al*., 2014; Soares Medeiros *et al*., 2017). Following the same approach, we applied CRISPR/Cas9 to disrupt the 69 kDa paraflagellar rod protein 2 gene (*BsPFR2*) and to test whether HDR occurred in *B. saltans*. The DSB was repaired with a donor DNA cassette containing *eGFP* fused with the drug selection gene *nptII/neo* and flanked by 500 bp of the untranslated regions (UTRs) upstream and downstream of the targeted *BsPFR2* as homologous repair arms.

## Materials and methods

### Source culture and culturing conditions

*Bodo saltans* Lake Konstanz (ATCC PRA‐428), kindly provided by Dr. Andrew P. Jackson (University of Liverpool, UK) for this study (Jackson *et al*., 2008 & 2016). *Bodo saltans* was cultured in ATCC Medium 802: Sonneborn's *Paramecium* medium, a cerophyl‐based medium enriched with 3.5 mM sodium phosphate dibasic (Na_2_HPO_4_). Cultures were incubated at 17°C and sub‐cultured weekly using new T‐25 vented tissue culture flasks (Falcon brand, Fisher Scientific) containing 30 ml of fresh media.

### Growth curve and kill curve of *B. saltans* in different antibiotics

We first tested the growth curve of *B. saltans* in the presence of different antibiotics (hygromycin, phleomycin, blasticidin). Cultures were diluted to 10^4^ ml^−1^, and 3 ml of the diluted cells were passed into each well of six‐well plates. Different concentrations of antibiotics were added to each well. The cells were cultured for 6 days in the presence of these different concentrations of antibiotics (ranging from 1 to 100 μg ml^−1^). The number of cells in each well were counted after 6 days. After these preliminary screenings, we chose 25 μg ml^−1^ hygromycin, 100 μg ml^−1^ of phleomycin and 100 μg ml^−1^ blasticidin for the kill curve experiments. Cells for kill curve experiments were cultured in T25 flasks with a starting density of 1 × 10^6^ ml^−1^. The culture media and antibiotics were changed every 4 days. The number of cells in each flask was counted on days 3, 7 and 15.

### sgRNA preparation and SaCas9 preparations

sgRNAs targeting *BsPFR‐2* (scaffold 1667WGS) were designed using the Eukaryotic Pathogen CRISPR Guide RNA/DNA Design Tool (http://grna.ctegd.uga.edu) with the SaCas9 option (21 bp target sequence preceding an NNGRRT PAM site). The sgRNAs were prepared as described previously (Peng *et al*., 2014; Soares Medeiros *et al*., 2017). We designed three forward PCR primers to generate DNA templates for each of the three sgRNAs (Supporting Information Table S1). Each primer is 99 bp long and consists of the T7 promoter sequence, the target sgRNA sequence that does not include the PAM sequence and the Scaffold Template annealing sequence. The reverse primer consists of the Guide‐it Scaffold Template (sequences of primers and the sgRNA are provided in Supporting information Table S1). The sgRNA DNA templates were amplified by PCR using Onetaq® 2X master mix with standard buffer (New England BioLabs), 30 μl of the sgRNA‐specific forward primer at a concentration of 10 μM and 18 μl of the SaCas9_sgRNA_R reverse primer at a concentration of 10 μM, and 2 μl of the sgRNA scaffold DNA template [double‐stranded DNA (dsDNA) from annealed and extended oligos, the SaCas9_scaff_F and SaCas9_scaff_R] with the following amplification conditions: 94°C for 2 min, 35 cycles of (94°C for 30 s, 66.5°C for 30 s, 68°C for 25 s) and a final elongation step at 68°C for 5 min. PCR products were purified using the E.N.Z.A. Cycle Pure kit following the manufacturer's instructions (Omega, BIO‐TEK). The purified PCR product was used for in vitro transcription using the TranscriptAid T7 High‐Yield Transcription Kit (ThermoFisher). We tested a range of concentrations of the purified sgRNA DNA template in the transcription reaction by using 500–2000 copies of the template DNA in 6 μl, 2 μl of each NTPs (ATP, UTP, CTP and GTP), 4 μl of 5X in vitro transcription buffer and 2 μl of TranscriptAid Enzyme Mix following established protocols (Peng *et al*., 2014; Soares Medeiros *et al*., 2017). The reactions were incubated for 5 h at 37°C in a preheated thermal cycler with a heated lid. The reaction was terminated by adding 15 μl of 3 M sodium acetate and 115 μl of nuclease‐free water. The sgRNA was precipitated by adding 2.5 volumes of 100% ethanol (375 μl) and placing the tube at −20°C overnight, followed by centrifugation at maximum speed (> 12,000*g*) at 4°C for 20 min. The supernatant was discarded, and the sgRNA pellet was washed by adding 75% and centrifuging again for 20 min at maximum speed (> 12,000 x *g*). All supernatants were removed carefully and the sgRNA pellet was air dried for > 20 min. The sgRNA was dissolved in 40 μl of nuclease‐free water and stored at −80°C. One microlitre of each sgRNA mixed with 18 μl of nuclease‐free water was used to visualize the sgRNAs on 2% gel agarose **(**Supporting Information Fig. S1).

### SaCas9 protein purification

*Staphylococcus aureus* Cas9 protein (SaCas9) was prepared following methods described by Soares Medeiros *et al*. (2017) with modifications. Briefly, the bacterial expression vector p6XHis_NLS‐SaCas9 (Addgene #101086) was transformed into *Escherichia coli* Rosetta 2(DE3) competent cells (Novagen) and grown as an overnight preculture with shaking at 37°C. Subsequently, 1 ml of preculture was used to inoculate 100 ml of terrific broth medium which was incubated with shaking at 37°C until it reached an optical density of 0.6 at 600 nm. Protein expression was induced by addition of isopropyl‐β‐d‐thiogalactopyranoside to a final concentration of 200 μM. Cells were grown overnight with vigorous shaking at 18°C. Following incubation, cells were collected by centrifugation, and the pellet was resuspended in 2 ml per 100 mg cell weight of xTractor Buffer (Takara Bio) plus 5 mg of lysozyme. Cells were lysed by sonication, and then 1 μl 2 ml^−1^ of DNAse I was added. The soluble fraction was purified using His60 Ni Superflow Resin & Gravity Columns (Takara Bio) following the manufacturer's recommendations. The concentration of SaCas9 containing fractions was determined by optical density at 280 nm and then the fractions were pooled. Pooled fractions were concentrated and desalted into Cytomix (120 mM KCl, 0.15 mM CaCl_2_, 10 mM KH_2_PO_4_, 25 mM Hepes, 2 mM EDTA, 5 mM MgCl_2_, pH 7.6) using Amicon Ultra‐15 centrifugal filter devices (Millipore Sigma). The protein concentration was determined using a BCA Protein Assay Kit (Pierce).

### SaCas9 and sgRNA assembly (ribonucleoprotein complex) and in vitro cleavage assay

We amplified *BsPFR‐2* from extracted DNA of wild‐type *B. saltans* cells. The PCR consisted of Onetaq® 2X master mix with standard buffer (New England BioLabs) and specific primers to amplify a 2 kb region of the *BsPFR‐2* gene (Supporting Information Fig. S2). Following PCR amplification, we purified the PCR products using the E.N.Z.A. Cycle Pure kit following the manufacturer's instructions (Omega, BIO‐TEK). In a 200 μl PCR tube, we combined 1 μg of each of three sgRNAs with 100 ng of SaCas9. We then incubated this mix in a thermo cycler at 37°C for 5 min. Following this incubation, we added 5 μl of the amplified and purified PCR product from above (amplified 2 kb of *BsPFR‐2*) to the sgRNA/SaCAS9 mix at a concentration of (300–600 ng μl^−1^), plus 1 μl of 15X BSA and 1 μl of nuclease‐free Duplex buffer [Cat#11‐05‐01‐12, Integrated DNA Technologies and adjusted the volume to 15 μl using RNase‐free water (Zymo Research, USA]. The tubes were incubated at 37°C for 1 h, followed by 5 min at 80°C to terminate the reaction and inactivate the ribonucleoprotein (RNP) complex. To visualize the cleaved DNA fragments by the sgRNA/Cas9 complex, we ran the entire 15 μl of each sample on a 2% agarose gel along with an appropriate DNA ladder and control samples (PCR products that were not treated by the RNP complex) (Supporting Information Fig. S2).

### Plasmid construction

We designed a 2512 bp cassette to target and knockout the *BsPFR2* gene (GenBank accession #CYKH01000743: scaffold1667, positions 3455 to 6406). This cassette is designed to replace *BsPFR2* with a fusion of the eGFP and neomycin genes. It contains 500 bp homologous arms at the 5′ and 3′ ends. These two homology regions flank the DSB site of the PFR2 gene and are intended to achieve precise repair of the DSB using the HDR mechanism. The construct was linearized with the restriction enzyme Xbal (New England BioLabs) prior to electroporation. The plasmid sequence was deposited in GenBank under accession number (MZ522125). The PFR2‐tomato Red Fluorescent Protein (RFP) construct was built by recombination PCR. The primers used are PFR2‐5'UTR‐F1 (TGCCTCAGAAACTGATGACG), PFR2‐3'UTR‐R1 (TTCGAATCCCCTCACTTCC), PFR2‐5′td‐R (CCCTAGGCGCCATGTTATGGTACTCTAAGG), PFR2‐td‐F (GTACCATAACATGGCGCCTAGGGTGAGCA), PFR2‐td‐R (GCATCGAAACACTTAGAGCTCGATATCGACGTCC) and Td‐PFR2‐3′‐F (ATCGAGCTCTAAGTGTTTCGATGCTTGTAGAAG). The generated construct was then cloned into the PCR‐blunt‐II Topo vector. The sequence of the construct was then verified by Sanger sequencing with M13 forward and reverse primers.

### Electroporation parameters and selection of transformants with neomycin

**Fig. 1:**
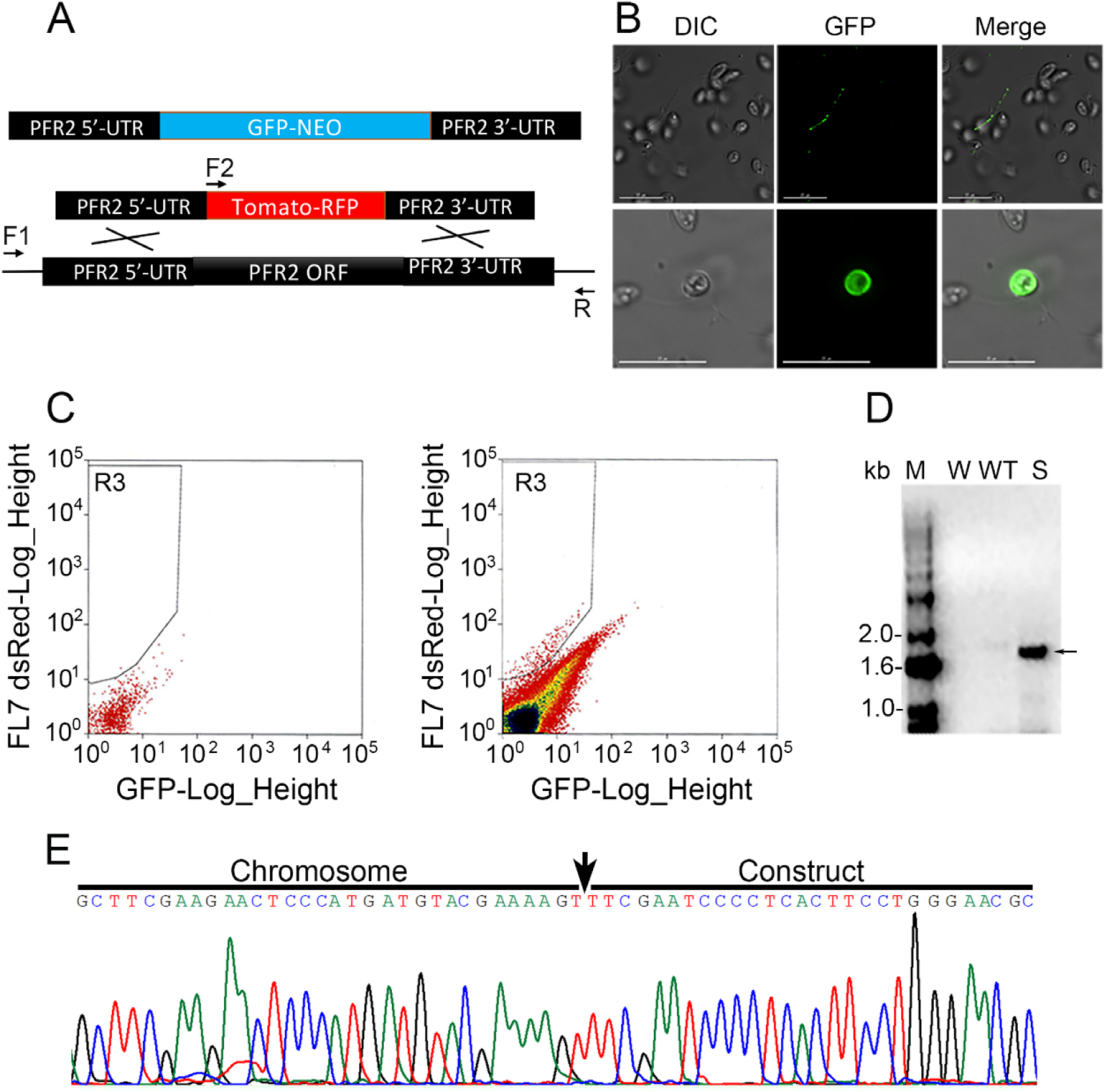
A. Diagram showing the eGFP‐NEO and tdTomato‐RFP constructs. A knockout construct can replace the endogenous *BsPFR2* with tdTomato by double cross‐over homologous recombination. The forward primers F1, F2 (against tdTomato) and reverse primer R were used for the semi‐nested PCR to detect the homologous recombination event. B. Live cell imaging of *eGFP‐NEO* transfected cells. *eGFP‐NEO* transfected cells have a strong phenotype. A free fluorescent flagellum (upper panel) and a fluorescent cell (bottom panel) are shown, scale bar 15 μm. The fluorescent cell has a strong motility defect (see Supporting Information Video S1). C. The fluorescent cells after transfection with the *PFR2‐tdTomato* construct were enriched by FACS. D. PCR products (F2 + R) were analysed by 1% agarose gel electrophoresis. Genomic DNA extracted from FACS sorted cells shows an amplicon of the expected size. M, DNA marker; W, water negative control; WT, wild‐type *Bodo saltans* cells; S, sorted *B. saltans* cells. E. Sanger sequencing of the purified PCR product confirmed the correct downstream genome sequence at the *BsPFR2* locus (that is not present in the introduced construct). Arrow indicates the start of the construct.

*Bodo saltans* cells were electroporated using a square wave electroporator (NEPA21, Bulldog Bio, Inc.), with the electroporation parameters presented in Table 1. Prior to electroporation, we premixed each of the RNP complexes that consisted of the sgRNAs each at a concentration 2–4 μg μl^−1^, SaCas9 at 100 ng μl^−1^ and the *PFR‐eGFP‐Neo* plasmid at a concentration of 5 μg μl^−1^. We added neomycin 2 days post‐electroporation at a concentration of 2 μg ml^−1^. We also use a BioRad electroporator with an exponential decay protocol set at 175 V, 500 μF and 400 Ω in 0.2 cm cuvette for experiments performed in Fig. 1 and Supporting Information Data S2. Pulse time was usually between 15 and 20 ms.

**Table 1:** NEPA 21 electroporation parameters used in our study.

	Poring pulse						Transfer pulse					
	*V*	PD (ms)	PI (ms)	*N*	Decay rate	Polarity	*V*	PD (ms)	PI (ms)	*N*	Decay rate	Polarity
1	200	25	0	1	10%	+	60	99	999	5	40%	+/−
2	99	5	50	7	10%	+	99	50	50	5	40%	+/−
3	150	5	50	3	10%	+	50	10	999	5	40%	+/−

V, voltage strength; PD, pulse duration; PI, pulse interval; N, number of pulses.

### Fluorescence‐activated cell sorting (FACS) to enrich transfected cells

*Bodo saltans* cells were collected by centrifugation and washed three times with distilled water to remove as many bacteria as possible. The final pellet was resuspended in 2 ml culture medium. After passing through the 35 μm mesh cap from Stellar Scientific (SKU: FSC‐9005) into a 5 ml FACS tube, the concentrated cells were used for sorting. We used 0.4X final sheath fluid for FACS of cells since they do not survive well in the normal 1X sheath fluid.

### Limiting dilution

To generate clonal cell lines from polyclonal transfected populations, we started by diluting cell pools that had been under selection with neomycin for 12 days. We transferred 500 μl of the transfected cells into Falcon tubes that contained 5 ml culture medium supplemented with neomycin at a concentration of 3 μg ml^−1^. Then, 100 μl of this mix was seeded into 48‐well plates; in total, we seeded four plates. The number of cells per plate well were between 50 and 100 cells per ml of cultured medium. Because *B. saltans* cells grow in suspension, we easily sub‐cultured these cell lines every 4 days by transferring 5 μl of the cell suspension to a new well containing 1 ml of culture medium and 3 μg ml^−1^ of neomycin.

### Phenotypic analysis by fluorescence microscopy

Emission of an eGFP signal by transfected cells was assessed and verified using a Zeiss Axio Imager M2 epifluorescence microscope (Carl Zeiss, Germany). The eGFP signal was detected in many, but not all clonal cell lines at the end of the 12 days (Fig. 3). Wells that contained bright transfected cells were used for genotyping. However, we noted that the fluorescence signal diminished over time, and 3 months post‐transfection, the eGFP signal could not be detected even though cell clones were still alive and maintained under neomycin selection. It is possible that the eGFP‐labelled cells died for reasons unknown at this time and were replaced by unlabelled cells resistant to neomycin.

### Genotyping for endogenous BsPFR‐2 knockouts

DNA was extracted from single populations of transfected cells recovered by limiting dilution and from wild‐type cells using the Quick‐DNA micro‐prep (Zymo Research, USA). We used PCR primers listed in Table 2 to amplify the *BsPFR2* locus and the flanking regions at both ends to assess whether *BsPFR2* was successfully disrupted and replaced by the DNA repair template and to confirm the correct genomic integration of the DNA repair template.

**Table 2:** PCR primers and their targeted loci on the PFR‐2 gene and the plasmid (DNA repair template) that were used in our study.

Primer ID	Sequence (5′ to 3′)	Targeted locus
PF1	GATTCAAGATCGATCTTCGAAC	*Bodo saltans* genome, 1.2 kb upstream the *PFR*‐2 gene
PF2	TGTAAAGCCCTTAGAGTACCAT	*B. saltans* genome and DNA repair template 30 bp upstream the *PFR*‐2 gene
PR3	TGCGCCTTGATGTAGAACTGCTC	*B. saltans* genome targets PFR‐2 gene at positions 645–667
PR4	ACATACAGACTTCCGCACT	*B. saltans* genome and DNA repair template 30 bp downstream the PFR‐2 gene
PR5	TATCGTGTAGAATGGTGTACTTGA	*B. saltans* genome, 0.8 kb downstream the *PFR*‐2 gene
PF6	CGTGATATTGCTGAAGAGCTTGGC	The neomycin (*neo*) gene on the DNA repair template
PR7	GCCAAGCTCTTCAGCAATATCACG	The neomycin (*neo*) gene on the DNA repair template
PR‐F1	GAGCTTCTGGAGACGAGAGC	*B. saltans* genome, 0.65 kb upstream the *PFR*‐2 ORF
PR‐F2	GTACCATAACATGGCGCCTAGGGTGAGCA	Tomato *RFP* 5′ open reading frame
PR‐R	CAGCAGACGCCGATTTCTCA	*B. saltans* genome, 0.72 kb downstream the *PFR*‐2 ORF

### RNA isolation and reverse transcription PCR confirmation for neomycin expression in *B. saltans*

Total RNA was isolated from 4 months old transformed clonal cell lines and from wild‐type *B. saltans* cultures following the same protocol described in Faktorová *et al*. (2020). The cDNA was synthesized using the QuantiTect Reverse Transcription Kit (Qiagen, Germany) according to the manufacturer's instructions. The reverse transcriptase (RT) Primer Mix and the 5X Quantiscript RT buffer were mixed in a 1:4 ratio in a 20 μl reaction that included 14 μl of RNA template (post‐DNA elimination). The cDNA was then amplified in a 25 μl PCR, with primers targeting the expression of the neomycin gene; *Neo F* and *Neo R* (Faktorová *et al*., 2020). Control PCRs were performed using RNA template without the RT to verify the absence of DNA from transformed *B. saltans* cells. PCR products were visualized by gel electrophoresis, with purified plasmid as a positive control for the PCR. Amplified PCR products at the expected size of 550 pb were documented (Supporting Information Fig. S5).

## Results

### Identification of selectable markers

In a previous study (Faktorová *et al*., 2020), we used neomycin as a selectable marker for genetic manipulation of *B. saltans*. To expand on the number of selectable markers, we tested several antibiotics that are widely used for genetic manipulation of *T. cruzi*, *Trypanosoma brucei* and related trypanosomatids and the results are in Supporting Information Data S1.

### Targeting PFR2 locus with fluorescent reporter genes using plasmid‐based approach

To demonstrate knockdown of expression of an endogenous gene in *B. saltans* we first targeted the paraflagellar rod 2 gene (*BsPFR2*) using a plasmid that contains a fluorescent protein to replace its open reading frame (ORF) by homologous recombination. The *PFR2* gene of *T. cruzi* was studied previously and was found to not be essential for growth, although knockout of *TcPFR2* has a phenotype (flagellar detachment) that can be detected by microscopy (Lander *et al*., 2015). *PFR2* has also been studied in *T. brucei* bloodstream forms and its disruption is lethal (Broadhead *et al*., 2006). As shown in Fig. 1A, two knockout constructs were created. We used ~ 500–600 bp of the coding sequence to flank the reporter genes *eGFP‐NEO* and *Td‐tomato‐RFP*.

Cells were first transfected with the *eGFP‐NEO* construct by electroporation. Two days after transfection, we collected the cells and performed live cell imaging. As shown in Fig. 1B, we found some strongly fluorescent cells (Fig. 1B, bottom panel). Interestingly, the fluorescent cells had a clear motility defect. As shown in Supporting Information Video S1, these cells barely moved in the culture medium. In some rare cases, we also found a free flagellum with weak eGFP signal (Fig. 1B, upper panel). This phenotype is consistent with the phenotypic changes observed for *T. cruzi* when *TcPFR2* was knocked out (Lander *et al*., 2015). The transfected cells were also selected with 3 μg ml^−1^ of neomycin starting from 48 h after transfection. Genomic DNA was extracted 2 weeks after transfection, and a positive signal detected by PCR with the primers targeting the *NEO* gene.

We also transfected *B. saltans* cells with a *td‐Tomato‐RFP* construct. FACS was used to enrich the red fluorescent cells. As shown in Fig. 1C, there was a subset of transfected cells with red fluorescence (Fig. 1C, right panel), but there was no fluorescence in cells transfected with buffer only (no DNA control) (Fig. 1C, left panel). The fluorescent cells were sorted and genomic DNA was extracted from these sorted cells and used as template for semi‐nested PCR to detect a homologous recombination event using the primers illustrated in Fig. 1A. As expected, we detected a specific expected PCR band in these sorted cells, but not in wild‐type control cells (Fig. 1D). The PCR product was gel‐purified and sequenced. The sequencing result (Fig. 1E) confirmed that the introduced construct had replaced the endogenous *PFR2* ORF by homologous recombination.

Several attempts to grow the sorted cells after transfection with both *eGFP‐NEO* and *tdTomato‐RFP* constructs were made. The sorted cells had a strong motility defect, and they were unable to grow. We believe that alterations of the flagellum have a stronger effect on *B. saltans* than on *T. cruzi* because *B. saltans* relies on its flagella to move and also to capture food. *Bodo* cells are likely not viable after losing their flagella. Additional attempts targeting the mitochondrial Ca^2+^ uniporter subunit c (MCUc) with a knockout construct with mNeon Green are described under Supporting Information Data S2. Transfection with a plasmid‐based approach for another gene with a higher expression level, the ribosomal 18S RNA gene, yielded similar results with low transfection efficiency. Despite achieving on‐target integration and maintenance for 2 weeks (Supporting Information Data S3), after a few weeks we were unable to detect the integrated plasmid in the ribosomal region using PCR. This result suggests excision of the plasmid from the genome occurred likely due to recombination between mutant and wild‐type alleles (Supporting Information Data S3) and that the rates of homologous recombination in *B. saltans* are very low and inefficient for relying on plasmid‐based approaches for manipulating the genome.

### SaCas9 RNPs in vitro specificity assay for the disruption of *B. saltans* *PFR2*

To disrupt *BsPFR2*, we used CRISPR/Cas9 gene editing for delivering RNP complexes composed of recombinant Cas9 from *Staphylococcus aureus* (SaCas9) and in vitro transcribed sgRNA.

**Fig. 2:**
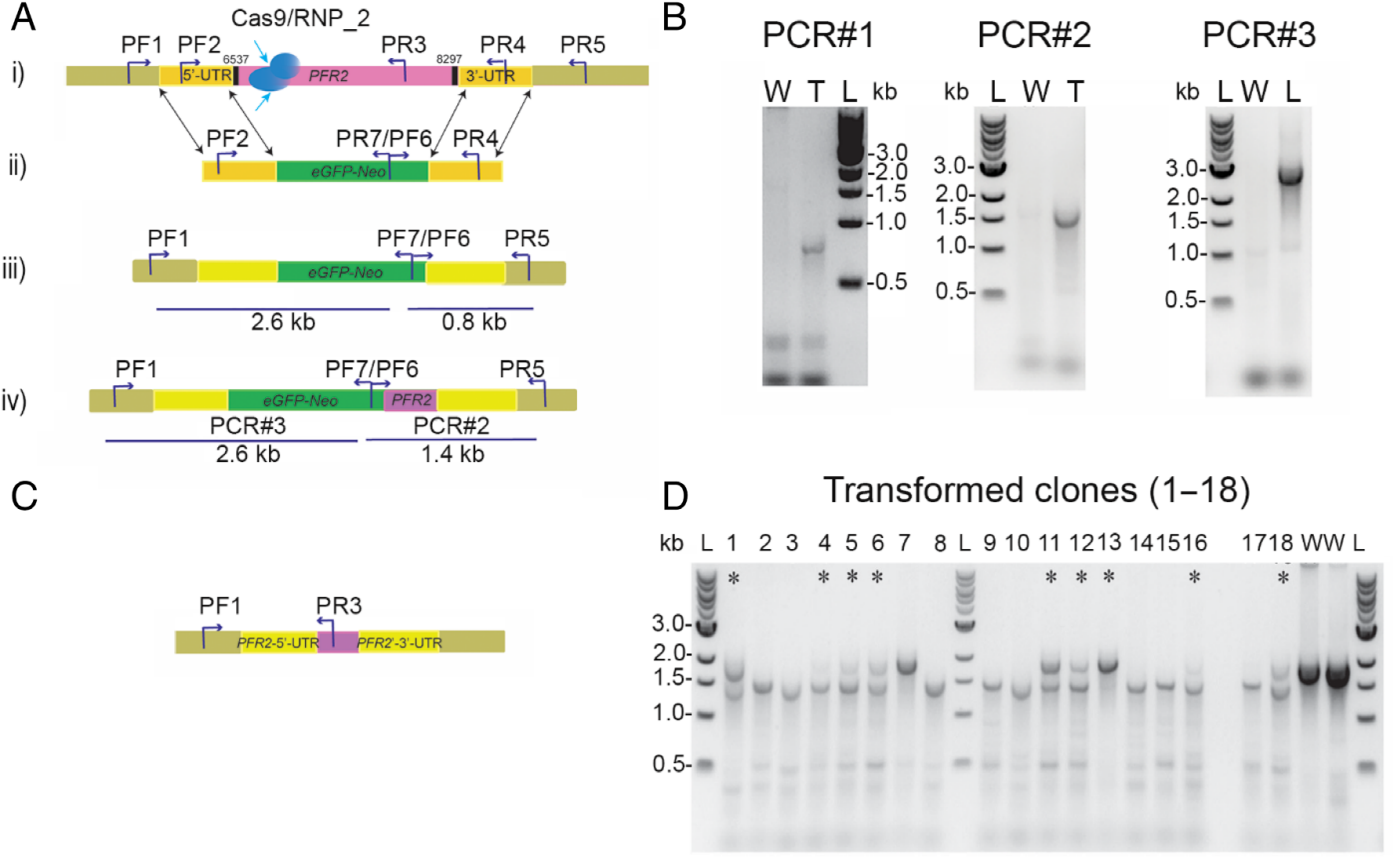
RNP2 transfection of *Bodo saltans*. A. Schematic representation of the strategy to generate *BsPFR2* mutants by CRISPR/Cas9‐induced homologous recombination: (i) a DSB was produced by SaCas9 and DNA was repaired with the eGFP‐NEO cassette containing 500 bp of the 5′ and 3’UTRs (ii). (iii) shows the replacement of the endogenous *BsPFR2* with eGFP‐NEO by double cross‐over homologous recombination; (iv) shows the replacement with an insertion of additional 600 bp of the *BsPRF2* resulting from a cross‐over event. PCR annealing sites are indicated in (i) to (iv). B. PCR analyses show that *BsPFR2* was ablated and replaced by either eGFP‐NEO (PCR#1, 0.8 kb) or by a cassette containing an additional insertion (PCR#2, 1.4 kb) as detected with primer PF6 and PR5 in (iii) and (iv). PCR#3 shows the replacement of the *BsPFR2* gene by eGFP‐NEO as detected with primers PF1 and PR7 (2.6 kb) in (iii) and (iv). L, 1 kb DNA ladder (Quick‐Load® 1 kb DNA Ladder, NEB), T, transfected cells, W, wild‐type cells. C. Schematic representation showing the replacement of the endogenous *BsPFR2* gene and the NHEJ repair mechanism. D. PCR products of amplified DNA from transformed clones 2 weeks post‐transfection and from wild‐type cells (W) with primer set PF1 and PR3. Wild‐type lanes (W) show bands of approximately 2 kb, the expected size of the amplified fragment of the *BsPFR2*. Transformed cells showing the homozygous (biallelic) or heterozygous (one disrupted allele, labelled with asterisks) knockout, with an upper band of 2 kb (corresponding to the size of the amplified fragment of wild‐type *BsPFR‐2*) and a lower band of 1.5 kb (corresponding to the size of the disrupted *BsPFR‐2* repaired by NHEJ).

We first tested the editing specificity of three RNP complexes of SaCas9 and sgRNAs in vitro to target different DNA loci of the *BsPFR2*. PCR products of 2 kb amplified from wild‐type *BsPFR2* (Fig. 2A) using primers PF1 and PR3 **(**Table 2) were incubated in vitro with each of three SaCas9 RNP complexes (Supporting Information Fig. S1). We observed that all three SaCas9 RNP complexes tested mediated cleavage of the targeted sequence of *BsPFR2*, whether used individually or in combination, as shown in Supporting Information Fig. S2. This result validates the potential utility of each of the SaCas9 RNP complexes for performing efficient genome editing upon delivery via electroporation into live *B. saltans* cells.

### Co‐delivery of SaCas9 RNPs and the DNA repair template‐mediated *BsPFR2* disruption

**Fig. 3:**
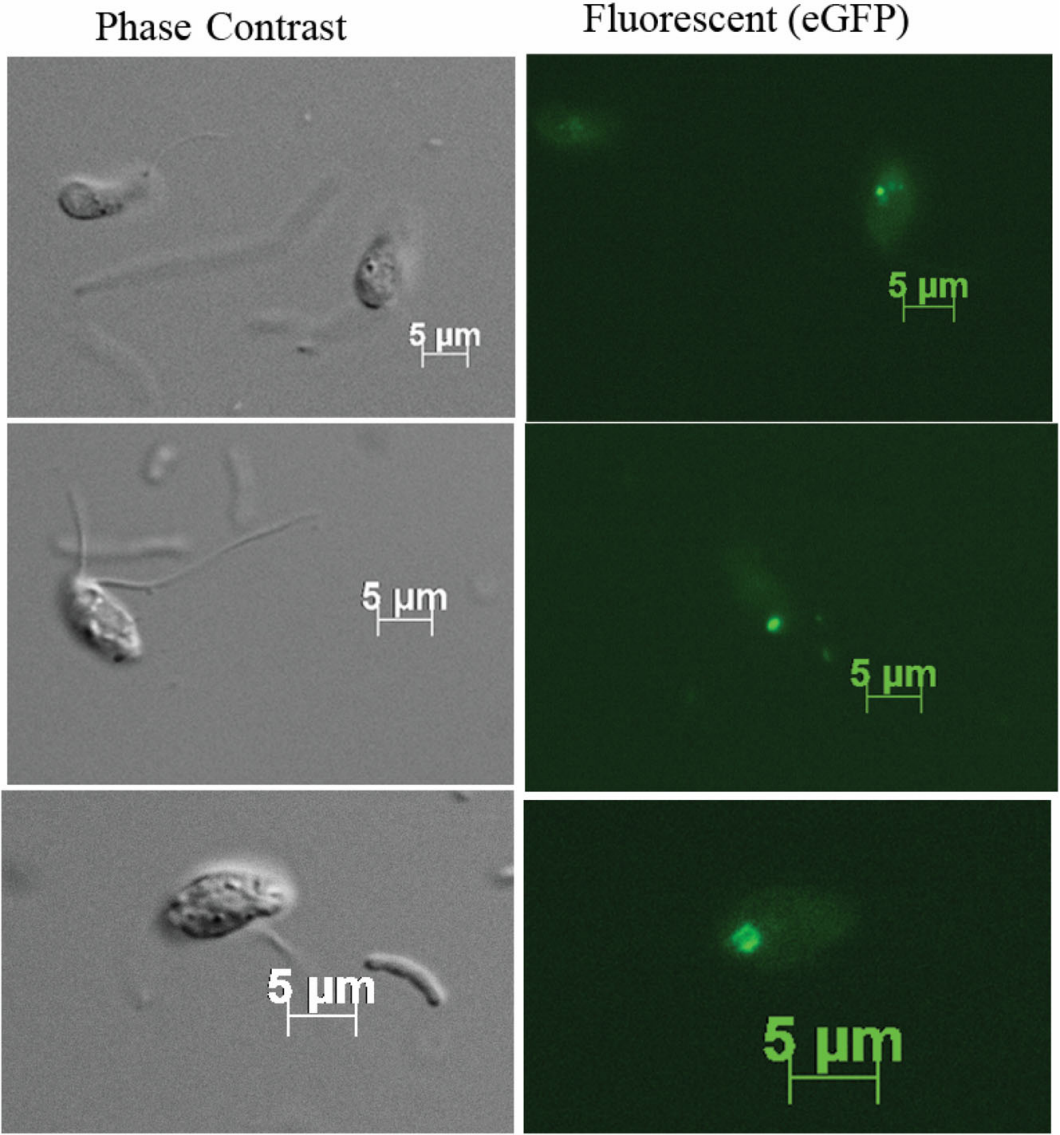
Phase‐contrast microscopy image (left), fluorescence microscopy image (right) showing the *Bodo saltans* transformed cells with eGFP signal. Scale bar = 5 μm.

To determine which of the SaCas9 RNP complexes provided a more successful outcome, we conducted independent transfection experiments with each of the three assembled SaCas9 RNP complexes delivered into the cells by electroporation along with the DNA repair template (Fig. 2A). Additional transfection experiments were conducted that co‐transfected all three RNP complexes together with the DNA repair template. We expected that using a combination of the three sgRNAs and the DNA repair template would increase the successful disruption of the endogenous *PFR2* gene and the efficiency of homologous recombination repair. The repair template consisted of dsDNA containing the fused *eGFP*, the neomycin resistance gene *nptII/neo* and 500 bp of homologous flanking sequences at both sides of *BsPFR2* gene to facilitate HDR (Fig. 2A). Neomycin was added to the transfected cell cultures 2 days post‐transfection at a concentration of 2 μg ml^−1^, and cells were sub‐cultured every 4 days into fresh medium supplemented with 2 μg ml^−1^ of neomycin. Cells from two transfection experiments survived selection: The first cell line that survived was one in which we used the SaCas9 RNP complex of sgRNA2 (here referred to it as ‘RNP2 transfection’; Figs 2 and 3); and the second, in which we used the three combined SaCas9 RNP complexes **(**here referred to it as ‘multiple RNPs transfection’; Supporting Information Fig. S3). Twelve days post‐transfection we performed clonal isolation by limiting dilution into 48‐well plates. We recovered multiple clones from these two transfected cell lines that were resistant to 2 μg ml^−1^ of neomycin, and we gradually increased neomycin to 3 μg ml^−1^ over 12 days.

Multiple clones from both RNP2 and multiple RNPs transfections lost their ability to swim and sunk to the bottom of the wells, and those non‐motile cells started to die gradually over the following 2–3 weeks post‐transfection (not shown). DNA was extracted from those clones during the 2–3 weeks prior to cell death, as well as from clones that maintained their ability to swim 4‐months post‐transfection. Genotyping analysis of clones obtained from the RNP2 transfection experiment using primer set PF1 and PR3 produced PCR products with molecular sizes of 1.5 and 2 kb (Fig. 2D). The 2 kb band corresponds to the wild‐type *BsPFR2* allele (Fig. 2A, i), while the 1.5 kb band corresponds to the disrupted *BsPFR2* alleles (Fig. 2C). Our results confirm the double knockout (homozygous) in clonal cell lines with PCR products that show bands of 1.5 kb (Fig. 2D), and single knockout (heterozygous) of *BsPFR2* in clonal cell lines that produced both 1.5 and 2 kb bands (Fig. 2D, lanes labelled with asterisks). Both single and double *BsPFR2* allele knockouts were detected in RNP2 transfection cell lines (Fig. 2D). Similar results were obtained from transfection experiments using multiple RNP complexes (Supporting Information Figs. S3A, i, iii and S3B). These data indicate that the Cas9 RNP complexes used in our study are efficient for inducing DSBs in target sequences.

In a few clones (4 colonies out of 18 colonies analysed) of the RNP2 transfection experiments we detected on‐target insertion of the DNA repair template (donor DNA) by homologous recombination (Fig. 2A, ii, iii). Genotyping analysis using primer sets that target the *Neo* gene localized on the DNA repair template and the flanking regions of the *BsPFR2* gene on the genome confirmed on‐target integration (Fig. 2B, PCR#1, Supporting Information Data S4). Moreover, in one of the clones from the RNP2 transfection, our sequence analysis showed that there was a switch in the 3′ flanking region between the repair template and the endogenous *PFR2* at the C‐terminus. This displacement resulted in an extension of an additional 600 bp at the 3′ flanking region (Fig. 2A, iv, PCR#2; Supporting Information Data S4). These results indicate that the repair of the DSB of the *PFR2* gene by homologous recombination generated non‐crossover events as well as crossover events (Fig. 2A, iii, iv).

Additional genotyping analysis of the same transfection with the Cas9RNP2 clonal cell line 3 months post‐transfection using primer sets targeting a fragment of the endogenous *BsPFR2* (PF1 and PR3) showed that the RNP2 clonal cell population still maintained one undisrupted *BsPFR2* allele, with PCR amplification producing fragment sizes corresponding to the *BsPFR2* gene of wild‐type cells at 2 kb (Supporting Information Fig. S4A) and a second DNA fragment with lower molecular size (1.5 kb) corresponding to the disrupted *BsPFR2* allele. Retention of one wild‐type *BsPFR2* allele allowed flagellar function, and hence survival of these cell lines, which were observed to have at least partial motility after 3 months post‐transfection. Moreover, our results confirmed the integration of the plasmid region that carries the neomycin gene in these surviving cell lines, using the primer sets PF6 and PR5 that anneal with the neomycin gene and the 3′ flanking region in the genome (Table 1; Supporting Information Fig. S4B). No integration of the other fragments of the plasmid that include the 5’ UTR or the *eGFP* was detected. The eGFP signal was not detected by fluorescence microscopy (not shown). This suggests these clonal cell populations survived because they retained an intact *BsPFR2* allele but they are also able to survive antibiotic selection by integrating a partial plasmid fragment that includes the neomycin gene through HDR at the 3′ flanking region (Supporting Information Fig. S4B, Data S4). The expression of the neomycin gene was confirmed by reverse transcription PCR (RT‐PCR) in these cultured cell lines that are still maintained in laboratory 4 months post‐transfection (Supporting Information Fig. S5).

In the transfection experiment using a combination of the three sgRNAs (Supporting Information Fig. S3), with a DNA repair template, our PCR and sequencing results obtained by screening clonal cell lines confirmed the disruption of the *BsPFR2* gene (Supporting Information Fig. S3C and D); however, we did not detect on‐target homologous recombination using the donor repair template in any clonal cell population from this multiple RNPs transfection experiment. Consistent with this, using plasmid targeted PCR primers (PF2 and PR4; Supporting Information Fig. S3D), sequencing confirmed that the plasmid was still maintained in the cells, likely off‐target or episomally, conferring resistance against the antibiotic selection (Supporting Information Fig. S3D). Sequence analysis of the lower 1.5 kb band suggested that the DSB was repaired by non‐homologous end joining (NHEJ) (Supporting Information Data S4, sequences labelled as PF1/PR3_KO_NHEJ**)**, with deletion of almost the entire *BsPFR2* gene. Among the seven clonal cell lines, we generated from the multiple RNPs transfection that were resistant to neomycin selection, there were five that were homozygous for the *BsPFR2* deletion (double knockout) that died within 2–3 weeks post‐transfection and at least one that was heterozygous for the deletion (single knockout) (Supporting Information Fig. S3B).

### *BsPFR2* is found to be essential for *B. saltans* growth and survival

Clones that had both *BsPFR2* alleles knocked‐out from transfections using only one SaCas9 RNP2 complex (Fig. 2D) or multiple SaCas9 RNP complexes (Supporting Information Fig. S3B) grew very slowly and died within 3 weeks of transfection, whether the repair template was integrated on‐target (Fig. 2B) or extra‐chromosomally (Supporting Information Fig. S3D). Other cell clones from both experiments that remained alive and showed growth rates comparable to the wild type showed only a single allele disruption, with the second *BsPFR2* allele remaining intact (Supporting Information Fig. S4A). Our results show that the strategy for repairing the DSB was achieved in *B. saltans* by two methods: HDR using the repair plasmid for on‐target integration (Fig. 2B), or by using the endogenous NHEJ repair mechanism (Fig. 2D, Supporting Information Fig. S3B).

## Discussion

In this study, we advanced genetic tool development for the kinetoplastid protist *B. saltans* using two approaches to knockout the *BsPFR2* gene; a plasmid‐based approach and CRISPR/Cas9 genome editing. The application of gene editing protocols for *B. saltans* is a prerequisite for deciphering many biological and ecological characteristics of this organism including the underlying mechanisms that support its wide distribution in both freshwater and marine environments, its stable association with bacterial symbionts (Midha *et al*., 2021), as well as its susceptibility to infection by one of the largest known viruses (Deeg *et al*., 2018). In addition, comparative genomics analysis to differentiate between the metabolic capacities of the parasitic trypanosomes and *B. saltans* identified candidate genes that contributed to the evolutionary transition to a parasitic lifestyle (Opperdoes *et al*., 2016). These genes could be promising targets for future CRISPR/Cas9‐based studies. As mentioned, successful transient transfection of a close relative of *B. saltans*, *Parabodo caudatus*, was demonstrated using plasmid‐based approaches that carried a eGFP gene; however, attempts to maintain stable transfection in *B. saltans* with complete plasmid integration into targeted loci were unsuccessful, and only partial on‐target integration of plasmid DNA was reported (Gomaa *et al*., 2017; Faktorová *et al*., 2020). Moreover, the transfection efficiency is very low, typically requiring several rounds of transfections to succeed. Similar challenges and constraints on achieving biallelic stable transgenics were reported in another kinetoplastid, *Trypanosoma cruzi*, a species with a similar genome structure to *B. saltans* (Jackson *et al*., 2008; Peng *et al*., 2014). Low rates of homologous recombination were observed for *T. cruzi* that required several weeks to confirm biallelic knockout and stable transfection (Lander *et al*., 2015; Soares Medeiros *et al*., 2017). CRISPR/Cas9 genome editing makes it possible to overcome some of the challenges of plasmid‐based transfection methods, by testing different guide RNAs and repair DNA templates to identify the combination that quickly and most efficiently induces biallelic mutation. CRISPR/Cas9 genome editing was used successfully to manipulate single and multicopy genes in *T. cruzi* (Peng *et al*., 2014).

Genome editing protocols using the CRISPR/Cas9 system have only been reported for a few marine protists; the choanoflagellate *Salpingoeca rosetta* (Booth and King, 2020),the marine diatom *Phaeodactylum tricornutum* (Stukenberg *et al*., 2018; Faktorová *et al*., 2020) and the protozoan parasite *Perkinsus marinus* (Yadavalli *et al*., 2021). Both plasmid‐based and CRISPR/Cas9 approaches were successful to knockout and replace the *BsPFR* in *B. saltans* with cassettes that carry a genetically fluorescent protein or a genetically fluorescent protein fused with an antibiotic resistance gene. NHEJ was found to be a common mechanism for repairing DSBs in *B. saltans*. Using CRISPR/Cas9 to induce a DSB potentially increases the likelihood of homologous direct repair using the donor DNA repair template.

We achieved on‐target plasmid integration by co‐delivering the DNA repair template with the RNP complex using only one sgRNA. However, we also detected that repair of the DSB can be achieved by NHEJ when one or multiple SaCas9 RNP complexes are transfected, even in the presence of the DNA repair template. In several clonal cell lines, we were able to confirm that single or double allele knockouts of the *BsPFR2* were achieved, inducing deletion of 500 bp of the *BsPFR2*. Sequence analysis confirmed that the lower band (1.5 kb) resulting from the DSB does not integrate the repair template through HDR. This instead reflects the loss of the PFR2 gene fragment targeted by the RNP complexes and suggests unknown determinants that biased the outcome toward long deletion of DNA before rejoining the blunt overhanging ends. The *B. saltans* DNA mechanism for repairing DSBs can be achieved by NHEJ as well as HDR.

Previous studies showed that phenotypic changes resulting from the knockout of the PFR‐1 and PFR‐2 genes in *T. cruzi* included flagellum detachment from the epimastigote cell body, leading to reduced cell motility, and growth defects (Lander *et al*., 2015). In addition, *PFR2* disruption is lethal in bloodstream forms of *T. brucei* (Broadhead *et al*., 2006). In *B. saltans* the disruption of both PFR‐2 alleles severely impacted cell motility and led to cell death within a couple of weeks. *B. saltans* is biflagellated, with one recurrent flagellum modified with hair‐like mastigonemes, that assists *B. saltans* to collect food particles by creating a current flow along the cell body toward the cytostome (Jackson *et al*., 2008). Therefore, loss of the ability to move/glide and to collect food particles properly will lead to cell starvation.

In conclusion, the genome‐editing protocols we developed for *B. saltans* for generating mutant strains with disrupted *BsPFR2* demonstrate CRISPR/Cas9 is a powerful tool for inducing DSBs, and suggest that both HDR and NHEJ are functional repair mechanisms in *B. saltans*. Successful methods for on‐target gene manipulation in *B. saltans* using our protocols can offer new advantages for studying gene function. Ribosomal genes for instance can serve as endogenous machineries for generating different gRNAs that can be used to target multiple genes involved in metabolic pathways of interest for elucidating their integrated/coordinated functions (e.g., symbiont recognition systems, salt tolerance genes). This study contributes to our expanding arsenal of tools for potential model organisms that will help us to advance understanding of the genomes and activities of protists.

## Conflict of interest

The authors declare no conflict of interest.

## Author contributions

V.E., R.D., F.G., Z.L. and P.R.G. conceived the study. F.G., Z.L. and D.B., performed the experiments. F.G., Z.L., H.A., R.D. and V.E. analysed and interpreted the data. F.G. and Z.L. composed the manuscript. All authors gave feedback on the manuscript.

## Supporting information


**Fig. S1**. Gel electrophoresis image.
**Fig. S2**. Cas9‐mediated cleavage of sgRNAs in vitro.
**Fig. S3**. RNP1, 2_3 (multiple RNP complexes) transfection of *Bodo saltans*
**Fig. S4**. A. RNP2 transfection of *Bodo saltans* 3‐month post‐transfection.
**Fig. S5**. RT‐PCR results confirming the neomycin gene expression in *Bodo saltans* transformants at the expected size of 550 bp.
**Table S1**. List of primers used for constructing sgRNAs.

**Data S1**. Identification of selectable markers.

**Data S2**. Targeting MCUc locus with fluorescent reporter protein.

**Data S3**. Supporting Information

**Data S4**. Sequences highlighted in yellow confirm the eGFP‐Neo‐PFR plasmid integration

Video S1.

## Data Availability

All the plasmids sequence used in this study are deposited in GenBank with accessions number provided in the manuscript. All protocols used in this study are deposited in Protocols.io under the following titles and DOI: Protocol for RNA isolation and RT‐PCR confirmation for Neomycin gene expression in transformed *Bodo saltans*.
dx.doi.org/10.17504/protocols.io.bxbxpipn
Protocol for Transfection of *Bodo saltans* with SaCas9 RNP complex in conjunction with *eGFP‐NEO* plasmid by electroporation.
dx.doi.org/10.17504/protocols.io.bxbwpipe
Protocol for sgRNA in vitro transcription and screening for effective SaCas9 RNP complex cleavage assay.
dx.doi.org/10.17504/protocols.io.bxbvpin6
SaCas9 protein purification.
dx.doi.org/10.17504/protocols.io.bxbupinw
Using FACS to sort fluorescent Bodo cells.
dx.doi.org/10.17504/protocols.io.skpecvn
Modified protocol to improve *Bodo saltans* yield in culture.
dx.doi.org/10.17504/protocols.io.9vyh67w
