# Supplementary material for: CRISPR/Cas9‐induced disruption of Bodo saltans paraflagellar rod‐2 gene reveals its importance for cell survival
Source: Environ Microbiol. 2022 Feb 2;24(7):3051–62. doi: 10.1111/1462-2920.15918 (PMC9544060; doi:10.1111/1462-2920.15918)
Supplement: Supplementary file 1 — Fig. S1. Gel electrophoresis image. Fig. S2. Cas9‐mediated cleavage of sgRNAs in vitro. Fig. S3. RNP1, 2_3 (multiple RNP complexes) transfection of Bodo saltans Fig. S4. A. RNP2 transfection of Bodo saltans 3‐month post‐transfection. Fig. S5. RT‐PCR results confirming the neomycin gene expression in Bodo saltans transformants at the expected size of 550 bp. Table S1. List of primers used for constructing sgRNAs. [file EMI-24-3051-s004.docx]

**Supplementary Table 1.** List of primers used for constructing sgRNAs. Target sequences included in the forward primers shown in blue, PAM sequences in red.

| Primer ID | Forward sgRNA primers including the sgRNA sequences | PAM |
| --- | --- | --- |
| SaCas9gRNA_PFRF1_KO | GAGAATTGTAATACGACTCACTATAGGGAGAGCGACGTCTTCAGCTTCAGATTGTTTTAGTACTCTGTAATTTTAGGTATGAGGTAGACGAAAATTGTA | GTGGAT |
| SaCas9gRNA_PFRF3_KO | GAGAATTGTAATACGACTCACTATAGGGAGAGCCAGTCGGACACGTGCAGGTCGTTTTAGTACTCTGTAATTTTAGGTATGAGGTAGACGAAAATTGTA | CTGGAT |
| SaCas9gRNA_PFRF4_KO | GAGAATTGTAATACGACTCACTATAGGGAGAGTTGCCGTGCTGAAGAACCTCGGTTTTAGTACTCTGTAATTTTAGGTATGAGGTAGACGAAAATTGTA | AGGAGT |
| SaCas9_sgRNA_R | AAAAAAATCTCGCCAACAAGTTGACGAGAT |  |
| SaCas9_scaff_F | GTTTTAGTACTCTGTAATTTTAGGTATGAGGTAGACGAAAATTGTACTTATACCTAAAATTACAGAATCTACTAAAACAAGGCAAAATGCCGTGTTTA |  |
| SaCas9_scaff_R | AAAAAAATCTCGCCAACAAGTTGACGAGATAAACACGGCATTTTGCCTTGTTTTAGTAGATTCTGTAATTTTAGGTATAAGTACAATTTTCGTCT |  |


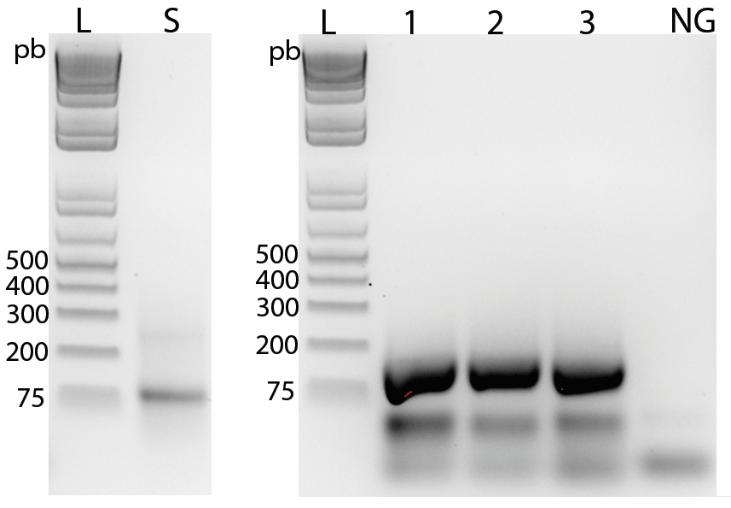


**Supplementary Figure 1**: Gel electrophoresis image showing the PCR product of the scaffold template (S) used to prepare the sgRNA (left panel), and gel electrophoresis of the three purified sgRNAs (lanes 1 to 3, right panel) after in vitro incubation with SaCas9 RNP complexes (1 to 3). Only 1 µl from each sgRNA was loaded into the gel; L, DNA ladder; NG, negative control.


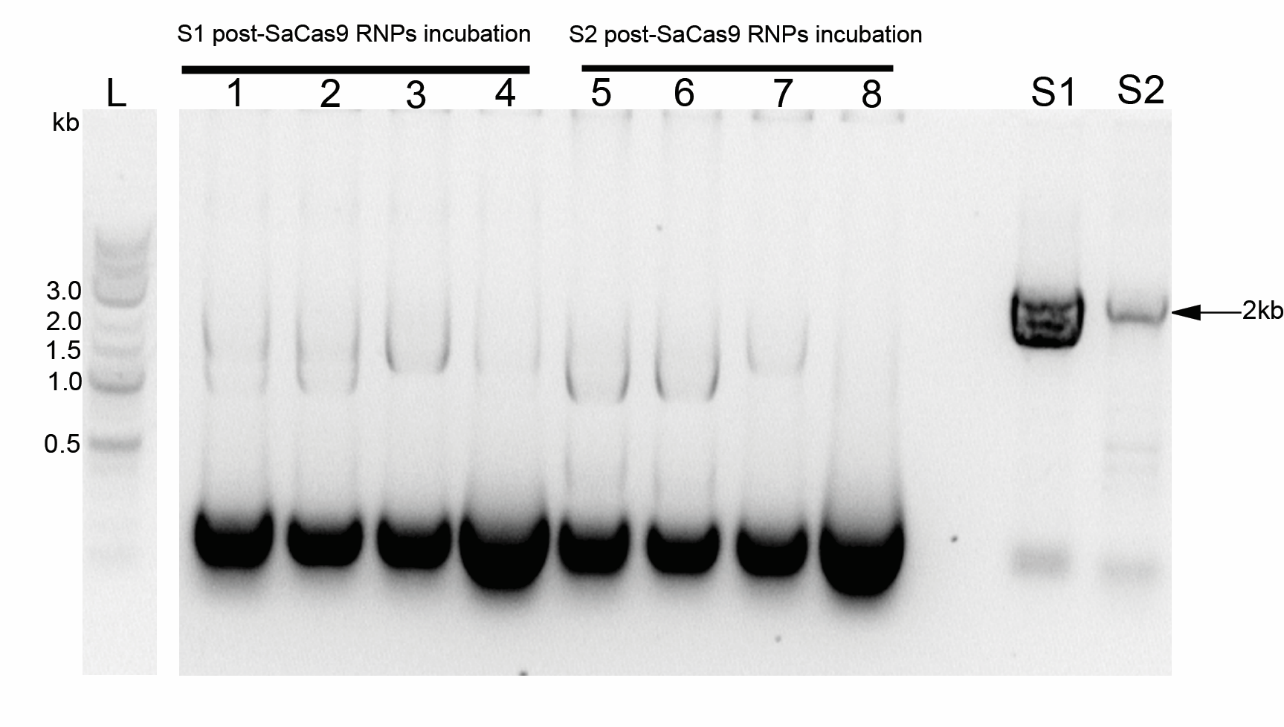


**Supplementary Figure 2**: Cas9-mediated cleavage of sgRNAs in vitro. The three synthesized sgRNAs directed Cas9-mediated cleavage of *BsPFR2* amplified fragment. Lanes S1 and S2 show the 2.8 kb amplified fragment of *BsPFR2* when primers PF1-PR3 were used. The differences between both S1 and S2 is the concentration of the amplified products that was used. Lanes 1 to 4 are the products of the S1 samples post in vitro cleavage assay with SaCas9 RNP1, 2, 3 and all the 3 SaCas9 RNPs complex combined, respectively. Lanes 5 to 8 are the products of the S2 samples post in vitro cleavage assay with SaCas9 RNP1, 2, 3 and all the 3 SaCas9 RNPs complex combined, respectively, also showing the fragments.


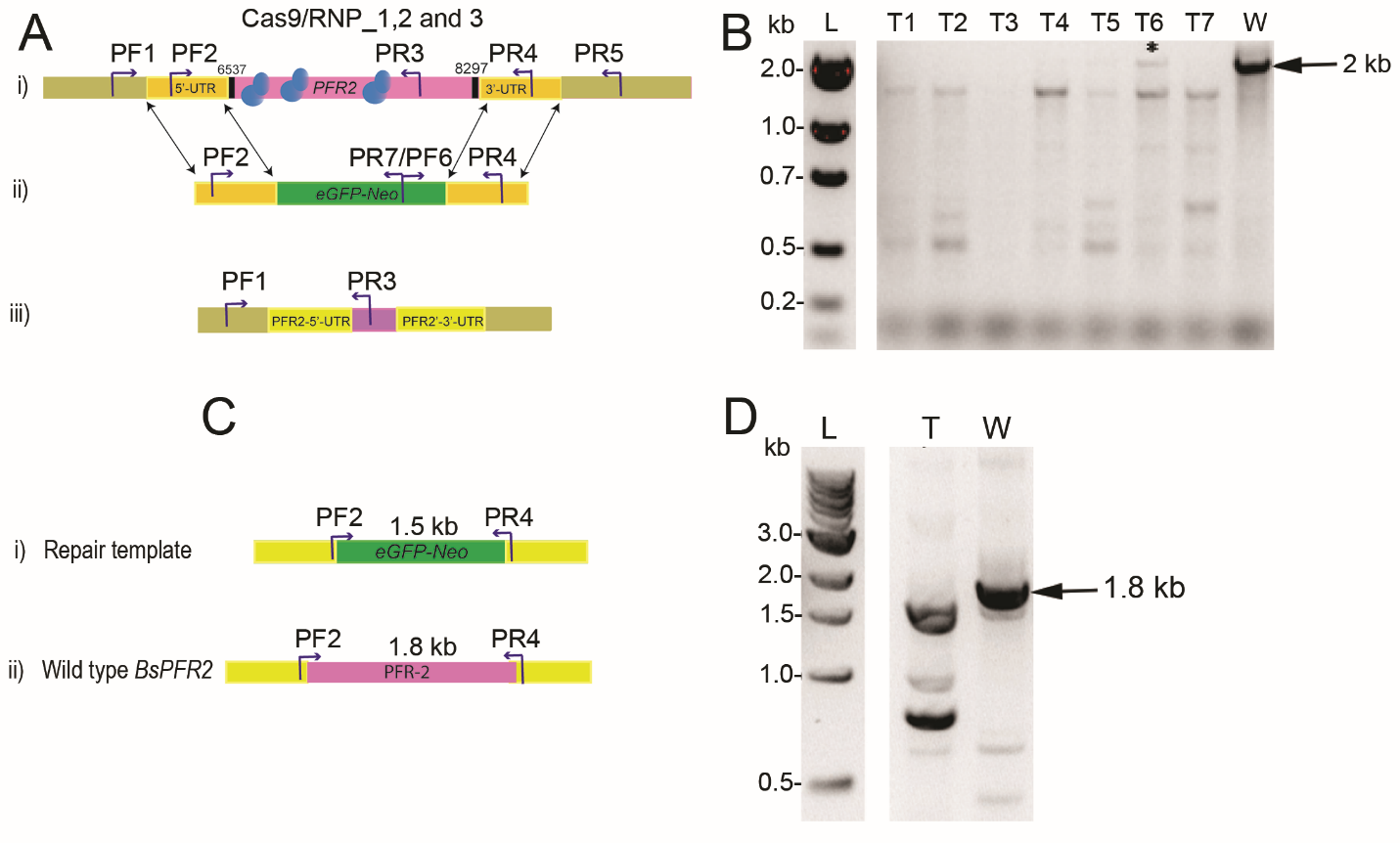


**Supplementary Figure 3: RNP1, 2_3 (multiple RNP complexes) transfection of *Bodo saltans****.* **A**. Schematic representation of the strategy to generate *BsPFR2* mutants by CRISPR/Cas9-induced homologous recombination: (i) a double strand break was produced at three sites by SaCas9, and DNA was repaired with the eGFP-NEO cassette containing 500 bp of the 5' and 3'UTRs (ii). (iii) shows the replacement of the *BsPRF2* resulting from a NHEJ event. PCR annealing sites are indicated in (i) to (iii). **B**. PCR analysis confirms homozygous (biallelic) or heterozygous (one allele, as shown in T6) knockout of *BsPFR2* in transfected cell clones (T1 to T7). An upper band of 2 kb corresponds to the size of the wild type *BsPFR2*, and a lower band of 1.5 kb corresponds to the size of the disrupted *BsPFR2*. Wild type lanes (W) show bands of approximately 2 kb, the expected size for the amplified fragment of *BsPFR2*. L, DNA ladder. **C.** The upper scheme shows the DNA repair template and the PF2 and PR4 primers annealing sites, the lower scheme shows the expected size of *BsPFR2* when amplified with the same primer set PF2 and PR4. **D**. PCR analysis showing the 1.5 kb DNA fragment amplified from transfected cells (T), and the 1.8 kb DNA fragment amplified from wild type cells (W).


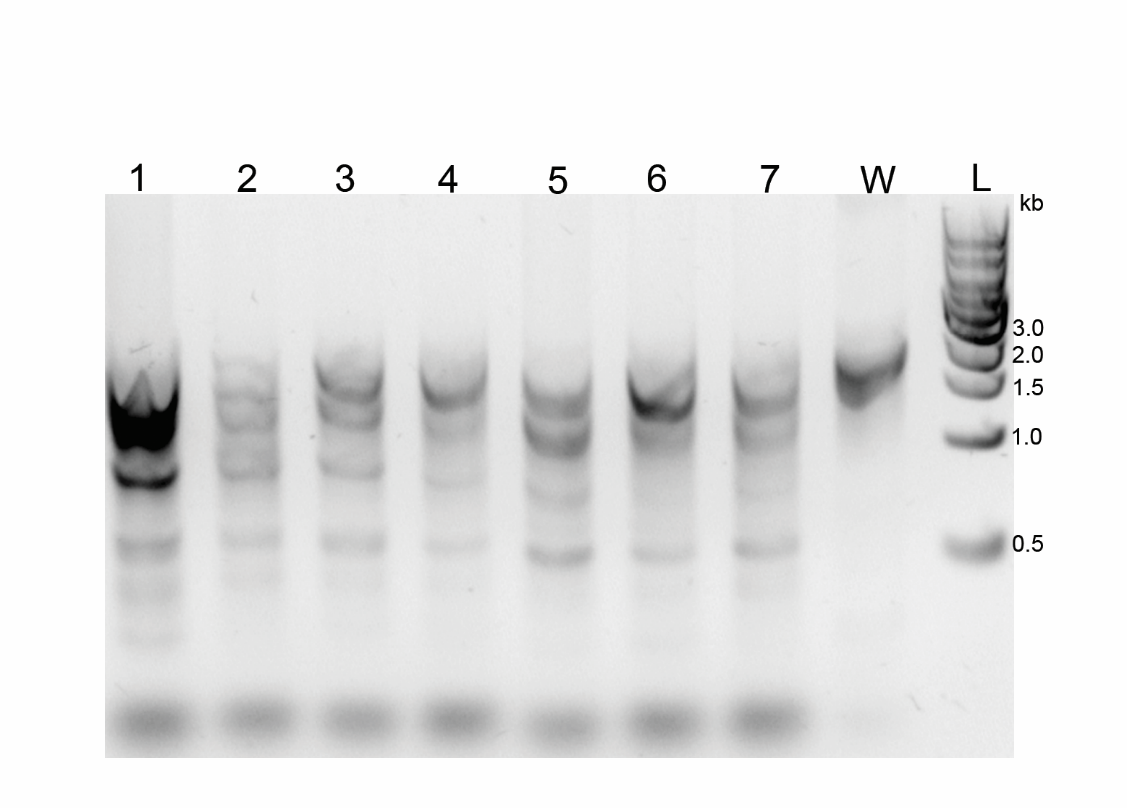


**Supplementary Figure 4A**: **RNP2 transfection of *Bodo saltans* 3-month post-transfection*.*** PCR products of amplified DNA from transformed clones using primers set PF1 and PR3, lanes 1 to 7, and wild type cells (W). Wild type cells (W) show a band of approximately 2 kb, the expected size of the amplified fragment of the *BsPFR2*. Transformed clone 3-months post-transfection, lanes 1- 7 showing 2 to 3 bands, the upper band corresponds to the intact *BsPFR2* allele while the second lower band at 1.5 kb is the disrupted PFR-2 allele. Note: The first few lanes are slightly tilted toward the left due to an error in gel preparation


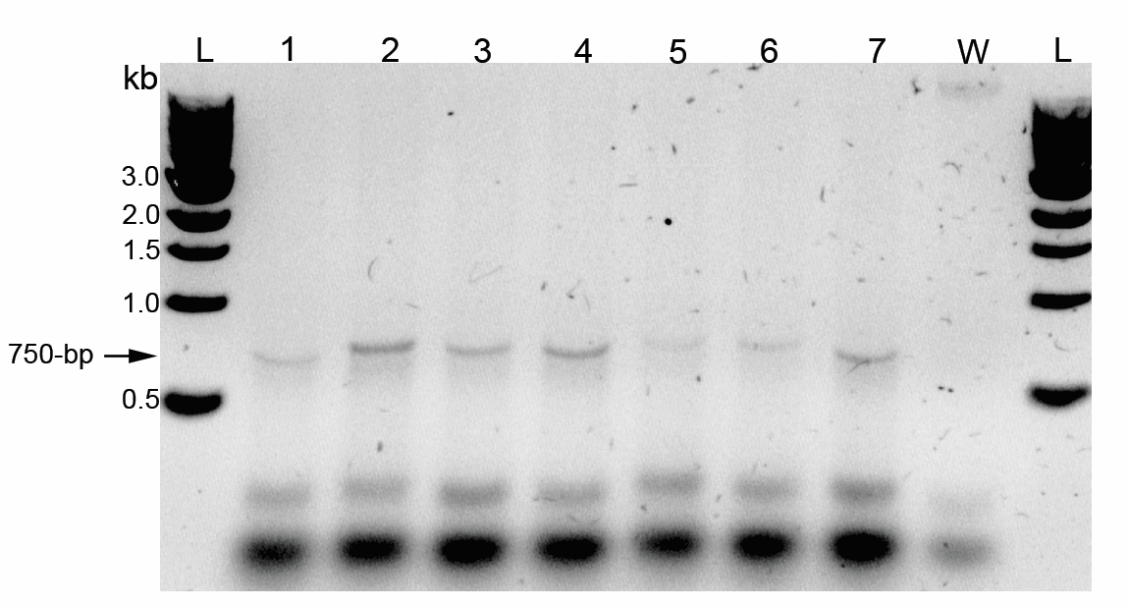


**Supplementary Figure 4B**: **RNP2 transfection of *Bodo saltans*** 3-month post-transfection PCR products of amplified DNA from transformed clones, and wild type cells (W) with primer set PF6 and PR5 (targeting both the Neomycin gene on the plasmid and the 3’ flanking region on the *B. saltans* genome). Wild type cells in lane (W) shows no band. The transformed clones 3 months post-transfection lanes 1-7 showing a band size at 750-bp confirms the plasmid integration into the genome. Sequence analysis of PCR products further confirms the correct integration.





**Supplementary Figure 5:** RT-PCR results confirming the Neomycin gene expression in *Bodo saltans* transformants at the expected size of 550- bp. L= 100 bp Ladder (NEB) ; lanes 1, Neomycin expression profile in *B. saltans* cells transformed with eGFP-Neo-PFR2 plasmid; lane 2, Control reaction using the same extracted RNA from transfected *B. saltans* without the RT step to verify the absence of DNA after the DNase treatments; lane 3, wild-type RT-PCR confirm the absence of the Neomycin expression in wild type samples; lane 4, control reaction using the same extracted RNA from wild-type *B. saltans* without the RT step to verify the absence of DNA after the DNase treatments; lane 5, PCR positive PCR control using the eGFP-Neo-PFR2 plasmid DNA; lane 6, PCR negative control. Forward and reverse PCR primers targeting the Neomycin gene are published in Faktorová et al. (2020). The PCR products were visualized under UV light using 1% agarose gel.
