# Supplementary material for: CRISPR/Cas9‐induced disruption of Bodo saltans paraflagellar rod‐2 gene reveals its importance for cell survival
Source: Environ Microbiol. 2022 Feb 2;24(7):3051–62. doi: 10.1111/1462-2920.15918 (PMC9544060; doi:10.1111/1462-2920.15918)
Supplement: Supplementary file 2 — Data S1. Identification of selectable markers. [file EMI-24-3051-s003.docx]

**Supplementary Data 1.**

**Identification of selectable markers.**

*B. saltans* cells were diluted to 10^4^ cells/ml and cultured for 6 days with different concentrations of antibiotics and the results are shown in Supplementary Data 1. Cells did not grow well at high concentrations of hygromycin (Data 1, Fig. 1A), phleomycin (Data 1, Fig. 1B) or blasticidin (Data 1, Fig. 1C) but they seem to tolerate puromycin, as they can still grow at 100 µg/ml, although at a much slower rate (Data 1, Fig. 1D).

The ideal concentration of a drug used for selection is the one that kills all wild type cells in 2 weeks. After preliminary testing with different concentrations of hygromycin, phleomycin and blasticidin, we chose 25 µg/ml hygromycin, 100 µg/ml of phleomycin, and 100 µg/ml blasticidin for our study. The kill-curve experiments are summarized in Supplementary Data 1E. Our results indicate that 25 µg/ml hygromycin, or 100 µg/ml of phleomycin are sufficient to kill all cells in less than 2 weeks. *B. saltans* is resilient to blasticidin, with very slow cell proliferation at 50 µg/ml or higher concentrations (Data 1, Fig. 1C). There were still ~10% of cells surviving after 2 weeks of cultivation with 100 µg/ml blasticidin **(**Data 1, Fig. 1C). We also tested 200 µg/ml blasticidin but some cells were observed alive after 2-weeks treatment. In summary, our results suggest that 25 µg/ml hygromycin and 100 µg/ml of phleomycin can be used for drug selection. 100 or 200 µg/ml blasticidin may also be used to enrich transfected cells since they are not able to grow under these conditions (Data 1, Fig. 1E).


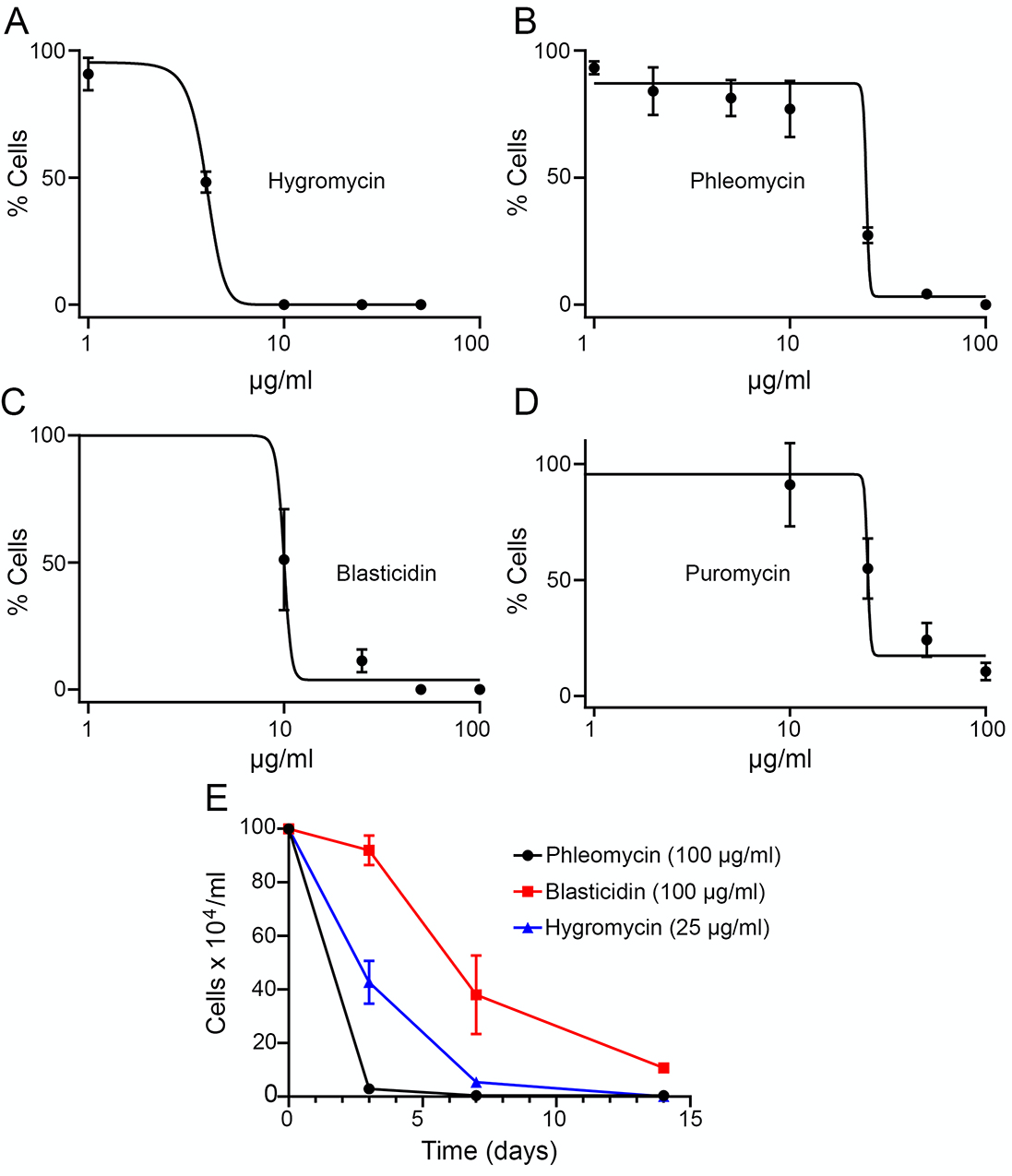


Data 1, Fig. 1. Inhibition of *B. saltans* growth by different antibiotics after 6 days. A, Hygromycin. B, Phleomycin. C, Blasticidin. D. Puromycin. Values are means ± s.d. from 3 independent experiments (n = 3). **E**. Antibiotics kill curve of 100 µg/ml of phleomycin, 100 µg/ml of blasticidin and 25 µg/ml hygromycin. Values are means ± s.d. from. 3 independent experiments (n = 3).
