# Supplementary material for: CRISPR/Cas9‐induced disruption of Bodo saltans paraflagellar rod‐2 gene reveals its importance for cell survival
Source: Environ Microbiol. 2022 Feb 2;24(7):3051–62. doi: 10.1111/1462-2920.15918 (PMC9544060; doi:10.1111/1462-2920.15918)
Supplement: Supplementary file 3 — Data S2. Targeting MCUc locus with fluorescent reporter protein. [file EMI-24-3051-s002.docx]

**Supplementary Data 2**

**Targeting MCUc locus with fluorescent reporter protein**.

MCUc (mitochondrial Ca^2+^ uniporter subunit c) has been studied in *T. cruzi* (Chiurillo et al., 2019) and *T. brucei* (Huang and Docampo, 2018). There was no growth phenotype when the expression of MCUc in both trypanosomes was downregulated. We designed a reporter knockout construct to knockout the *BsMCUc*. We cloned both 5’-UTR and 3’-UTR of *B. saltans* MCUc to flank the reporter gene mNeon Green by recombination PCR. The primers used in this study are MCUc-5UTR-F (TTGACGCTGATTCCTTCCTT), MCuc-5UTR-4Neon-R (CTTGGACACCATGAAACCACACAACCTGAGCAAAC), MCUc-Neon-F (GTTGTGTGGTTTCATGGTGTCCAAGGGCGAGGA), MCUc-Neon-R (GGTACAAAGCTATTACTTGTACAGCTCGTCCATG), MCU-3UTR-F-4Neon: CTGTACAAGTAATAGCTTTGTACCTTCCCTCAATA, MCUc-3UTR-R (TCGATGCTTGTGATCTCCTG). The final PCR product was then cloned in pCR-blunt-II-topo vector (Data 2, Fig. 1A). We amplified the knockout cassette with M13 forward and reverse primers from the vector and used the purified PCR product for transfection. Two days after transfection, we did FACS sorting to enrich the green cells. The sorted cells were grown for 8 days, and then collected for a 2^nd^ sorting. As shown in Data 2, Fig. 1C-E, there were very low percentage of green cells during the 1^st^ sorting, but there were much higher percentage of green cells in the 2^nd^ sorting (from 0.01% to 0.27%, 26-fold increase). This result suggests that FACS sorting is a useful tool to enrich transfected cells. The genomic DNA was extracted from the wild type and sorted cells. We used the 3 primers shown in Data 2, Fig. 1A (F: CAAGCGGCGTCGACAATTAC, R1: ACGTACATCGGCTGGTTCTT and R2: GGTAGGTCTTCTTGGAGCGG) to detect whether there was any homologous recombination event. As shown in Data 2, Fig. 1B, we detected a specific band with the size consistent with the predicted homologous recombination event. The PCR product was purified and directly sequenced. As shown in Data 2, Fig. 1F, the sequencing result was consistent with the predicted homologous recombination event.


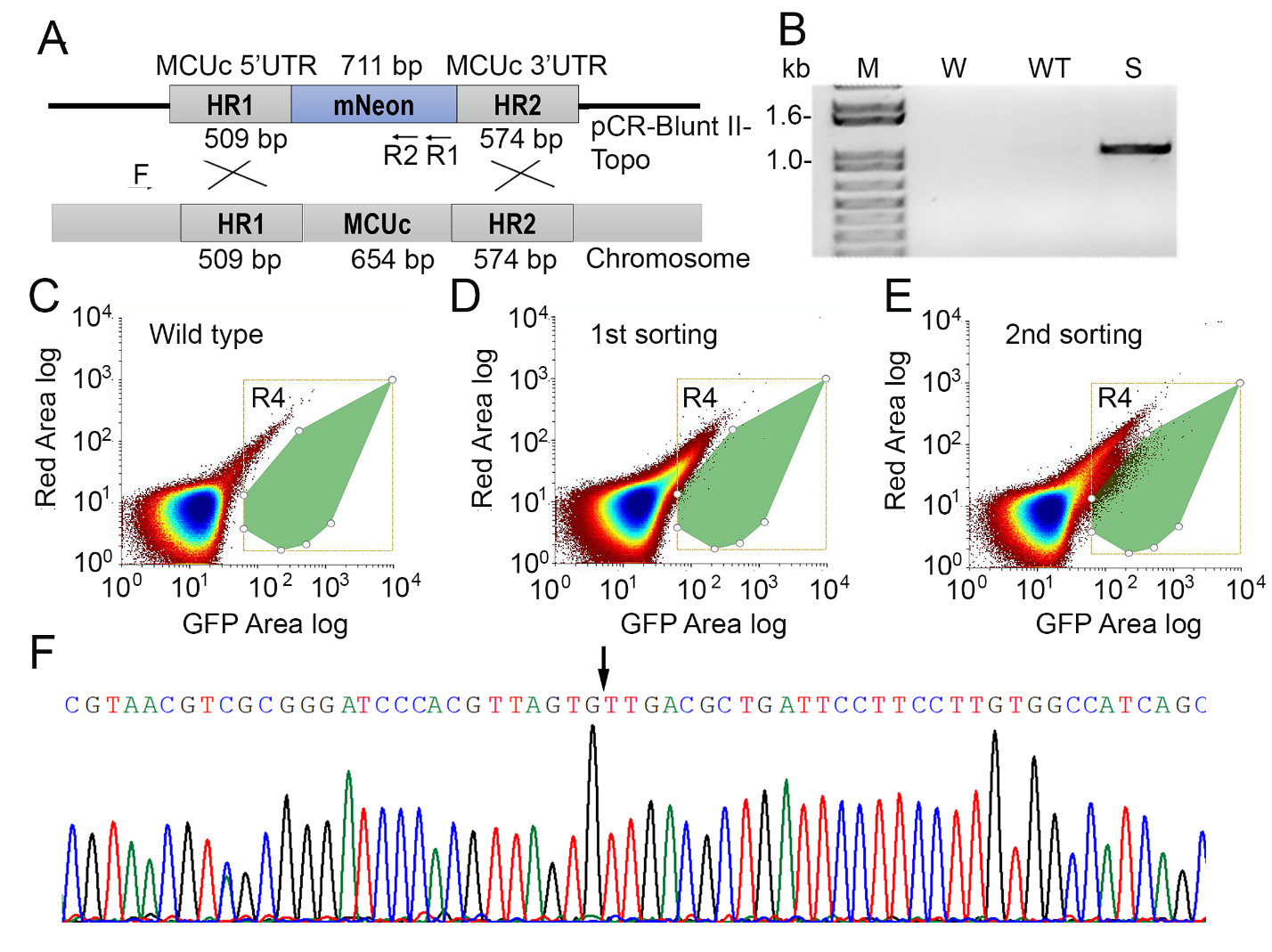


Data 2, Figure 1. A, Scheme showing the MCUc-mNeonGreen construct. This construct is expected to replace the endogenous MCUc gene with mNeonGreen by double crossover homologous recombination. The Primers F, R1 and R2 were used to detect homologous recombination. B, The semi-nested PCR products (F+R2) were analyzed by 1% agarose gel electrophoresis. C-E, FACS sorting of the MCUc-mNeon transfected cells. C. FACS sorting of control wild type cells. D. first sorting of MCUc-mNeon transfected cells. E, 2nd sorting of the MCUc-mNeon transfected cells that had been enriched by FACS. F, Sanger sequencing of the purified PCR product. The sequencing confirmed the correct upstream genome sequence before MCUc locus.
