# Supplementary material for: CRISPR/Cas9‐induced disruption of Bodo saltans paraflagellar rod‐2 gene reveals its importance for cell survival
Source: Environ Microbiol. 2022 Feb 2;24(7):3051–62. doi: 10.1111/1462-2920.15918 (PMC9544060; doi:10.1111/1462-2920.15918)
Supplement: Supplementary file 4 — Data S3. Supporting Information [file EMI-24-3051-s001.docx]

**Supplementary Data 3**

On-target integration of the 18S-GFP-Neomycin plasmid (Data 3, Fig. 1A) which carries 1,000 bp of the *Bodo saltans* small subunit ribosomal RNA gene was achieved for at least one copy of the 18S rRNA gene. This plasmid was maintained under selection in *B. saltans* cultures for approximately three weeks, after which it was excised by the wild-type allele. This plasmid is designed to integrate within the small subunit ribosomal RNA gene region of *B. saltans*. It carries two homologous arms, each 500-bp of the *B. saltans* small subunit ribosomal RNA gene. In order to construct this cassette, we first had to sequence the complete ribosomal operon of *B. saltans*, which has a total length ̴9 kb. The structure of the ribosomal operon of *B. saltans* is similar to the closely related kinetoplastida *Leishmania*, where the ribosomal RNA genes are arranged in head- to-tail repeats (Data 3, Fig. 1B), (Yan et al (1999). For the cassette construction we used only 1 kb region of the 18S ribosomal RNA gene, which is represented in the scheme as 500-bp Homologous Recombination Region 1 (HR1) and 500-bp Homologous Recombination Region 2 (HR2), two genes, the GFP and Neo genes which are located in the middle of the construct and their expression is controlled by the presence of the tubulin intergenic region (Bs TUB IGR) at the 3’ and 5’ of each gene. Since transcription in *B. saltans* is polycistronic, the presence of the tubulin intergenic region is necessary for *trans*-splicing and polyadenylation (Plasmid sequence was submitted to GenBank accession number MZ522126). We transfected *B. saltans* cells with the 18S-GFP-Neomycin cassette and selected transfected cells using 3 µg/ml of G418. We obtained confirmation of correct integration based on gel electrophoresis images after PCR using 6 sets of primers (Data 3, Figs 1c and 2), and sequencing of the PCR products.


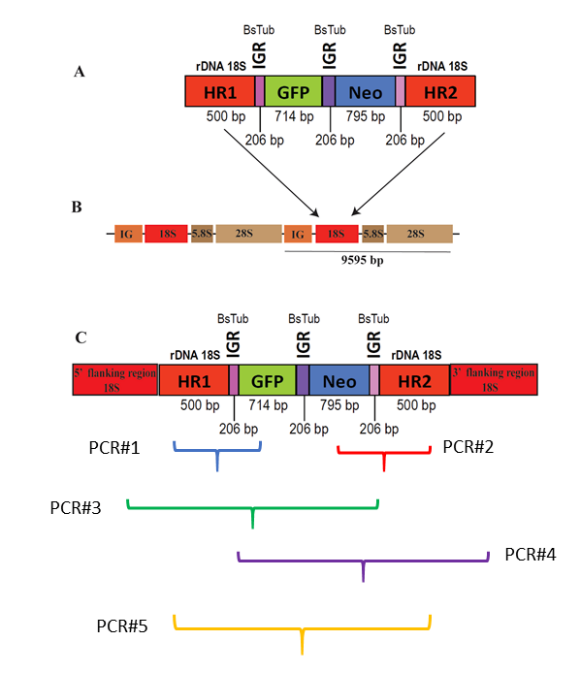


**Data 3, Figure 1**. A. Scheme of the 18S-GFP cassette containing the *B. saltans* tubulin intergenic regions (IGR), *GFP*, *Neo* and 500 bp homologous regions (HR1 and HR2) from the 18S locus. B. Ribosomal operon in the *B. saltans* genome. C. Plasmid integration in *B. saltans* genome through HR1 and HR2. PCR primers that were used are indicated.

Gel electrophoresis (Data 3, Fig. 2) shows amplified PCR products for each primer set at the expected sizes. PCR primers and sequences are shown in Data 3, Table 1. PCR amplifications using control, or wild-type cells, are noted with “C.” PCR #1 uses primer set HR1 forward & GFP reverse and an 800 bp product is expected. PCR #2 uses primer set Neo forward & HR2 reverse and a 1000 bp product is expected. Both PCRs 1 and 2 are internal to the cassette and confirm the presence and the expected orientation of genes within the cassette. PCR #3 uses primer set BsTubR IGR & flanking1forward, and this primer pair set confirms integration of the cassette at the correct location within the 18S operon by capturing one of the flanking regions of the 18S rRNA gene as well as the integrated cassette. There are 3 copies of the same tubulin intergenic region (BsTub IGR) at three different locations in the 18S-GFP cassette and the first and the third copies have an identical sequence, while the second copy (middle) intentionally has a slightly different sequence. The BsTubR IGR primer that we designed amplifies the first and the third copies. We therefore expect 2 bands of different sizes (600 bp and 2200 bp) and this is observed in PCR #3. PCR #4 with primer set BsTub IGR_forward & flanking 2 reverse is used to confirm integration at the correct position by capturing the opposing flanking region of the 18S rRNA gene and the cassette. As above, we expect 2 bands of different sizes due to the specificity of the BsTubR IGR primer to amplify the first and third copies (850 bp and 3.6 kb). The additional bands in PCR#3 and PCR#4 are probably due to unspecific binding with the middle copy of the tubulin IGR (BsTub).

PCR #5 uses primer set HR1 forward & HR 2 reverse, and this primer pair captures most of the cassette sequence. Transfected cells exhibit two bands, 350 bp and 2800 bp, which represent the presence of both the wild type ribosomal 18S gene and the integrated cassette.

**Data 3, Figure 2**. PCR products of two separate samples of *B. saltans* cells transfected with the 18S-GFP Cassette (S1 and S2) and wild type cells (C). Five different primers sets were used to amplify and confirm the plasmid integration into the genome as illustrated in Data 3, Figure 1C: Primer set #1 targeted the HR1 of the plasmid and the GFP gene, primer set# 2 targeted the HR2 of the plasmid and Neomycin gene, primer set #3 targeted the tubulin IG region and the 5’ UTR flanking region in the genome, primer set #4 targeted the tubulin IG region and the 3’UTR flanking region in the genome, primer set #5 targeted both HR1 and HR2 of the plasmid, the upper band (2.7 kb) obtained from plasmid amplification, while the lower band (300 bp) is the 18S region amplified from the wild type gene.

Data 3, Table 1: List of primers used to generate the sequence data for the 18S cassette transfection

| Primer IDs | Sequence 5'->3' |
| --- | --- |
| HR1- Forward- tagging | CGATGAGGCAGCGAAAAGAAATAGG |
| HR2- Reverse- tagging | CATGCATGACATGCGTGAATCGGAAA |
| Tubulin IG-Forward | TTCGCATTCTTGGGTCGGCGCGCTGTGTTT |
| Tubulin IG-Reverse | CTCTTCCGAGACGGTGTGTACATCGCACC |
| GFP_18S_Reverse | AGTTGTATTCCAATTTGTGTCCAAGAATGTTT |
| Neo_18S_Forward | CGCTTGGGTGGAGAGGCTATTCGGCTATGACT |
| Flanking _genome_Forward | TTGATTCTGCCAGTAGTCATATGCTTGTT |
| Flanking _genome_Reverse | TCGGGATTCTTGTTCTCACTGACATTGT |

PCR sequences obtained from transfected *B. saltans* with SSU-GFP-Neo plasmid, sequences in red confirm the plasmid integration at both ends. PCR#5 sequences are not included here.

>18SGFP Plasmid

GAACCAATCGGACGTGCTCCATTCACGCGGCGTTCGGGCTTGCCCGGGCGACGTAGCGAGTCGTCCAGCGAATGAATGAAAGTAAAACCAATGCCGCGGACGCGGCAGCAGCACTCAGAAGTGTTGACTCAATTCATTCCGTGCGAAAGCTGGTCTAACCAGCGTATTTTGACGAACAACTGCCCTATCAGCCAGTGATGGCCGTGTAGTGGACTGCCATGGCGTTGACGGGAGCGGGGGATTAGGGTTCGATTCCGGAGAGGGAGCCTGAGAAATAGCTACCACTTCTACGGAGGGCAGCAGGCGCGCAAATTGCCCAATGTCGATAAAAACGATGAGGCAGCGAAAAGAAATAGGCCTCCCAGCCACTCTGTGGTTGGGAGATACAATGGGGGATATTTAACGCCATCCAAAATCGAGTAACAATTGGAGGACAAGTCTGGTGCCAGCACCCGCGGTAATTCCAGCTCCAAAAGCGTATATTAACGCTGTTGCTGTTAGTGCGATGTACACACCGTCTCGAAAGAGAACACTTGTGCGTTAGAGCGCTTGGCTGATGTGTTCGCATTCATGGGTCGGCTCGCTGTGTTTCTGTATCGATGCACTTGCCGCGTGCTGCTATGCCATGCCCTCTCCTCTCTTTTCTTCTTTTCTCACTGCATACTAACAGAAAAGAAAAGCTACCACTCTTCAAACCACAAAAGAACATGAGTAAAGGAGAAGAACTTTTCACTGGAGTTGTCCCAATTCTTGTTGAATTAGATGGTGATGTTAATGGGCACAAATTTTCTGTCAGTGGAGAGGGTGAAGGTGATGCAACATACGGAAAACTTACCCTTAAATTTATTTGCACTACTGGAAAACTACCTGTTCCATGGCCAACACTTGTCACTACTTTCACTTATGGTGTTCAATGCTTTTCAAGATACCCAGATCATATGAAACGGCATGACTTTTTCAAGAGTGCCATGCCCGAAGGTTATGTACAGGAAAGAACTATATTTTTCAAAGATGACGGGAACTACAAGACACGTGCTGAAGTCAAGTTTGAAGGTGATACCCTTGTTAATAGAATCGAGTTAAAAGGTATTGATTTTAAAGAAGATGGAAACATTCTTGGACACAAATTGGAATACAACTATAACTCACACAATGTATACATCATGGCAGACAAACAAAAGAATGGAATCAAAGTTAACTTCAAAATTAGACACAACATTGAAGATGGAAGCGTTCAACTAGCAGACCATTATCAACAAAATACTCCAATTGGCGATGGCCCTGTCCTTTTACCAGACAACCATTACCTGTCCACACAATCTGCCCTTTCGAAAGATCCCAACGAAAAGAGAGACCACATGGTCCTTCTTGAGTTTGTAACAGCTGCTGGGATTACACATGGCATGGATGAACTATACAAATAGCGCACGCTCGTTGTGTAGTGGTGCGTGAGGAGGAAGTGTTTTTGTGTCCATTGCTGCACTGGCGTGGTTGGCCGTTGGCGACCCCAGCTTGCTTCCACTGCCCTTGCGGGGGCGTGGCTGCTACGTTGGGGCGCCGCCTCGCGCGTTGCTGCGTGCGCTGGACGCTGCGCAATCATATGATGTACGTAGAGGTGTGCGTCATCGACATGATCTCCGCTGTTTTCCGCTGTTTTGTTTGTGCCGTCTTGTCTTCCATGATTTGTCCCTCTTCCATTTGTTTGACTGCTTTTGACCACCCAACACAAACAGAGACATAGCGAAGGAGAGCATGCTTGAACAAGATGGATTGCACGCAGGTTCTCCGGCCGCTTGGGTGGAGAGGCTATTCGGCTATGACTGGGCACAACAGACAATCGGCTGCTCTGATGCCGCCGTGTTCCGGCTGTCAGCGCAGGGGCGCCCGGTTCTTTTTGTCAAGACCGACCTGTCCGGTGCCCTGAATGAACTGCAGGACGAGGCAGCGCGGCTATCGTGGCTGGCCACGACGGGCGTTCCTTGCGCAGCTGTGCTCGACGTTGTCACTGAAGCGGGAAGGGACTGGCTGCTATTGGGCGAAGTGCCGGGGCAGGATCTCCTGTCATCTCACCTTGCTCCTGCCGAGAAAGTATCCATCATGGCTGATGCAATGCGGCGGCTGCATACGCTTGATCCGGCTACCTGCCCATTCGACCACCAAGCGAAACATCGCATCGAGCGAGCACGTACTCGGATGGAAGCCGGTCTTGTCGATCAGGATGATCTGGACGAAGAGCATCAGGGGCTCGCGCCAGCCGAACTGTTCGCCAGGCTCAAGGCGCGCATGCCCGACGGCGAGGATCTCGTCGTGACCCATGGCGATGCCTGCTTGCCGAATATCATGGTGGAAAATGGCCGCTTTTCTGGATTCATCGACTGTGGCCGGCTGGGTGTGGCGGACCGCTATCAGGACATAGCGTTGGCTACCCGTGATATTGCTGAAGAGCTTGGCGGCGAATGGGCTGACCGCTTCCTCGTGCTTTACGGTATCGCCGCTCCCGATTCGCAGCGCATCGCCTTCTATCGCCTTCTTGACGAGTTCTTCTAGGTGCGATGTACACACCGTCTCGGAAGAGAACACTTGTGCGTTAGAGCGCTTGGCTGATGTGTTCGCATTCTTGGGTCGGCGCGCTGTGTTTCTGTGCCGATGCACTTGCCGCGTGCTGCTATGCCATTCCTCTCCTCGCTTTTCTTCTTTTCTCACTGCATACTAACAGAAAAGAAAAGCTACCACTCTTCAAACCACAAAAGAACAAGCAAGTCCCGCCCACTTCGGGTTGGTGACCCTTGACCTTGCGGTTCGTGAACATTGATACAAGAAGCACGGGAGTGGTTCCTTTCCGATTCACGCATGTCATGCATGCCGGGGGGCGCCCGTGATTTTTACTGTGACTAAAGAAGCGCGACCAAAGCAGTCATTCGACTTGAATTAGAAAGCATGGGATAACAAAGGAGCAACCTACGGTACTACCGTTTTCGGCTTTTGTTGGTTTTAGAAGTCTGTGGGAGATTATGGTTTCGTGCGACCGGCGGCTGGGGTCCGTCCCCGCGCTTTTTGCTTTGTGGGGTTCGCCTTGCATTGTAGACTCGCGAGACCAGGAATGAAGGAGGGTTGTTCGGGGGAAAACGTACTGGGGCGTGAGAGGTGAAATTCTTAGACCGCCCCAAGACGAACTACAGCGAAGGCATTTTCCAAGGATACCTTCCTCAATCAAGAACCAAAGTGTGGAGATCGAAGAT

>Genomic ribosoomal 18S

CGTAATCTGCCGCAAAAATCTTGCGGTCTCCGCATTATTGGATACCTTGGCGAAACGCCAAGCTAATACATGAACCAATCGGACGTGCTCCATTCACGCGGCGTTCGGGCTTGCCCGGGCGACGTAGCGAGTCGTCCAGCGAATGAATGAAAGTAAAACCAATGCCGCGGACGCGGCAGCAGCACTCAGAAGTGTTGACTCAATTCATTCCGTGCGAAAGCTGGTCTAACCAGCGTATTTTGACGAACAACTTGGGGGGNGGGAACNGGTCGATTCCGGANGGGAGCCTGAGAAAAGCTACCACTTCTACGGAGGGCAGCAGGCGCGCAAATNGCCCAATGTCGATAAAAACGATGAGGCAGCGAAAAGAAATAGGCCTCCCAGCCACTCTGTGGTTGGGAGATACAATGGGGGATATTTAACGCCATCCAAAATCGAGTAACAATTGGAGGACAAGTCTGGTGCCAGCACCCGCGGTAATTCCAGCTCCAAAAGCGTATATTAACGCTGTTGCTGTTAAAGGGTTCGTAGTTGAACTGAGGGCCGCCAAGCGCACAAGCAAGTCCCGCCCACTTCGGGTTGGTGACCCTTGACCTTGCGGTTCGTGAACATTGATACAAGAAGCACGGGAGTGGTTCCTTTCCGATTCACGCATGTCATGCATGCCGGGGGGCGCCCGTGATTTTTACTGTGACTAAAGAAGCGCGACCAAAGCAGTCATTCGACTTGAATTAGAAAGCATGGGATAACAAAGGAGCAACCTACGGTACTACCGTTTTCGGCTTTTGTTGGTTTTAGAAGTCTGTGGGAGATTATGGTTTCGTGCGACCGGCGGCTGGGGTCCGTCCCCGCGCTTTTTGCTTTGTGGGGTTCGCCTTGCATTGTAGACTCGCGAGACCAGGAATGAAGGAGGGTTGTTCGGGGGAAAACGTACTGGGGCGTGAGAGGTGAAATTCTTANACCGCCCCAGACNAACTACANCGAAGGCATTTTCCAAGGATACCTTCCTCAATCAAGAACCAAAGTGTGGAGATCNAANATGATTANAGACCATTGTANTCCACACCACAAACGGTGACACCCATGAATNGGGGAATACTTTGGTTGCCTT

>PCR_1

CCGAAAGAAATAGGCCTCCCAGCCACTCTGTGGTTGGGAGATACAATGGGGGATATTTAACGCCATCCAAAATCGAGTAACAATTGGAGGACAAGTCTGGTGCCAGCACCCGCGGTAATTCCAGCTCCAAAAGCGTATATTAACGCTGTTGCTGTTAGTGCGATGTACACACCGTCTCGAAAGAGAACACTTGTGCGTTAGAGCGCTTGGCTGATGTGTTCGCATTCATGGGTCGGCTCGCTGTGTTTCTGTATCGATGCACTTGCCGCGTGCTGCTATGCCATGCCCTCTCCTCTCTTTTCTTCTTTTCTCACTGCATACTAACAGAAAAGAAAAGCTACCACTCTTCAAACCACAAAAGAACATGAGTAAAGGAGAAGAACTTTTCACTGGAGTTGTCCCAATTCTTGTTGAATTAGATGGTGATGTTAATGGGCACAAATTTTCTGTCAGTGGAGAGGGTGAAGGTGATGCAACATACGGAAAACTTACCCTTAAATTTATTTGCACTACTGGAAAACTACCTGTTCCATGGCCAACACTTGTCACTACTTTCACTTATGGTGTTCAATGCTTTTCAAGATACCCAGATCATATGAAACGGCATGACTTTTTCAAGAGTGCCATGCCCGAAGGTTATGTACAGGAAAGAACTATATTTTTCAAAGATGACGGGAACTACAGACCACGTGCTGAAGTCCAGTTTGAAGGTGATACCCCTGTTGATAGAAATCGAGTTAAAAG

>PCR_1

GGGNCCCGGAAAAGAAATAGGCCTCCCANCCNCTCTGTGGTNGGGAGATACAATGGGGGATATTTAACGCCATCCAAAATCGAGTAACAATTGGAGGACAAGTCTGGTGCCAGCACCCGCGGTAATTCCAGCTCCAAAAGCGTATATTAACGCTGTTGCTGTTAGTGCGATGTACACACCGTCTCGAAAGAGAACACTTGTGCGTTAGAGCGCTTGGCNGATGTGTTCGCATTCATGGGTCGGCTCGCTGTGTTTCTGTATCGATGCACTTGCCGCGTGCTGCTATGCCATGCCCTCTCCTCTCTTTTCTTCTTTTCTCACTGCATACTAACAGAAAAGAAAAGCTACCACTCTTCAAACCACAAAAGAACATGAGTAAAGGAGAAGAACTTTTCACTGGAGTTGTCCCAATTCTTGTTGAATTAGATGGTGATGTTAATGGGCACAAATTTTCTGTCAGTGGAGAGGGTGAAGGTGATGCAACATACGGAAAACTTACCCTTAAANTTATTTGCACTACTGGAAAACTACCTGTTCCATGGCCAACACTTGTCACTACTTTCACTTATGGTGTTCAATGCTTTTCAAGATACCCAGATCATATGAAACGGCATGACTTTTTCAAGAGTGCCATGCCCGAAGGTTATGTACAGGAAAGAACTATATTTTTCAAAGATGA

>PCR_1

CCGGNGGANGTAGCGANTCTTCNGGGAANGAATGAANNTAAAACCNATGNCGGGGACGCGNCAGCAGCANTCAGAAGTGTTGACTCAATTCATTCCGTGCGAAAGCTNNTCTANCCAGCGTATTTTGACGAACAACTGCCTTATCAGCCAGTGAGTCCGTGTAGTGNANTGNCANGGCGNTGANNNGAGCGGGGGGANTAGGTTCGATTCTNGAGAGGGAGCCTGAGAAATAGCTACCACTTCTACGGAGGGCAGCAGGCGNGCAAATTGCCCAATGTCGATAAAAANGATGAGGCAGCGAAAAGAAATAGNCCTCCCAGCCACTCTGTGGTTGGGAGATACAATGGGGGATNTTTNACGCCNTCCAAAATNGAGTANCAATTGGAGGACAAGTNNGGTGCCAGCACCCGCGGTAATTCCAGCTCCAANAGCGTATATTAACGCTGTTGNTGTTAGTGCGATGTACACACCGTCTCGAAAGAGAACACTTGTGCGTTAGAGCGCTTGGCTGATGTGTTCGCATTCATGGGTCGGCTCGCTGTGTTTNTGNATCGATGCNCTTGCCGCGTGNTGNTATGCCATGCCCTCTCCTCTCTTTTNTTCTTTTCTCAGTGCATNCTANCAGAAAAGAAAAGCTACCACTCTTCANACCACAAAAGAACATGAGTAAAGGAGAAGAACTTTTCACTGGAGTTGTCCCAATTCTTGTTGAANTAGATGGTGATGTTAANGGGCACAAATTTTCNGTCNGTGGAGAGGGTGAAGGTGATGCAACATNCGGAAAACTTACCNTTAAATTTATNTGCACTCNTGGAAAACTACCTGTTCCATGNCCAACACTTGTCACTACTTTCACTTATGGTGTNCAATNCTTTTCAAGATACCCAGATCATATGAAACGGCATGACTTTTTCAAGAGTGCCATGCCCGAAGGTTATGTAGGAAAGATATTTTTCAAAGATGACGNGAACNACAAGAC

>PCR_2

TTAGCCCAAGGGNGCCNGGGTTCCTTTTGATAAGACCGACCTGTCCGGCGCCCTGAATGAACTGCCGGACGAGGCAGCGCGGCTATCGTGGCTGGCCACGACGGGCGTTCCTTGCGCAGCTGTGCTCGACGTTGTCACTGAATCGGGAAGGGACTGGCTGCTATTGGGCGAAGTGACCGGGCAGGATCTCCTGTCATCTCACCTTGCTCCTGCCGAGAAAGTATCCCTCCTGGATGATGCGATGCGGCGGCTGCCTACGCTTGATCCGGCTACCTGCCCATTCGACCACCAAGGGAAACATCACATCGAGCGAGCACGTACTCGGATGGAAGCCGGTCCTGTCCATCAGGATGATCTGGACGAAGAGCATCAGGGGCTCGCGCCAGCCGAAATGTTCGCCAGGTTCAAGGCGCGCATGCCCGACGGCGAGGATCTCGTCTTGACCCATGGCGATGCCTGCTTGCCGAATATCATGGTGGAAAATGGCCGCTTTTCTGGATTCTTCGACTGTGGCCGGCTGGGTGTGGCGGACCGCTATCATGACATAGAGTTGGCTACCCGTGATATTGCTGAAAATCTTGACGGCGAATGGGCTGACCGCTTCCTCGTGCTTTACGGTATCGCCGCTCCCGATTCTCAGCGCATCGACTTCTATCGCCTTCTTGACGAGTTCTTCTAGGCGCGATGTACACACCGTCTCGGAAGAGAACACTTGTGCGTTAGAGCGCTTGGCTGATGTGTTCGCATTCTTGGGTCGGCGCGCTGTGTTTCTGTGCCGATGCACTTGCCGCGTGCTGCCTTGCCATTCCTCTCCTCGCTTTTCTTCTTTTCTCACTGCATACTAACAGAAAAGAAAAGCTACCACTCTTCAAACCACAAAA

>PCR_2

GTCAGCGCAGGGGCGCCCGGTTCTTTTTGTCAAGACCGACCTGTCCGGTGCCCTGAATGAACTGCAGGACGAGGCAGCGCGGCTATCGTGGCTGGCCACGACGGGCGTTCCTTGCGCAGCTGTGCTCGACGTTGTCACTGAAGCGGGAAGGGACTGGCTGCTATTGGGCGAAGTGCCGGGGCAGGATCTCCTGTCATCTCACCTTGCTCCTGCCGAGAAAGTATCCATCATGGCTGATGCAATGCGGCGGCTGCATACGCTTGATCCGGCTACCTGCCCATTCGACCACCAAGCGAAACATCGCATCGAGCGAGCACGTACTCGGATGGAAGCCGGTCTTGTCGATCAGGATGATCTGGACGAAGAGCATCAGGGGCTCGCGCCAGCCGAACTGTTCGCCAGGCTCAAGGCGCGCATGCCCGACGGCGAGGATCTCGTCGTGACCCATGGCGATGCCTGCTTGCCGAATATCATGGTGGAAAATGGCCGCTTTTCTGGATTCATCGACTGTGGCCGGCTGGGTGTGGCGGACCGCTATCAGGACATAGCGTTGGCTACCCGTGATATTGCTGAAGAGCTTGGCGGCGAATGGGCTGACCGCTTCCTCGTGCTTTACGGTATCGCCGCTCCCGATTCGCAGCGCATCGCCTTCTATCGCCTTCTTGACGAGTTCTTCTAGGTGCGATGTACACACCGTCTCGGAAGAGAACACTTGTGCGTTAGAGCGCTTGGCTGATGTGTTCGCATTCTTGGGTCGGCGCGCTGTGTTTCTGTGCCGATGCACTTGCCGCGTGCTGCTATGCCATTCCTCTCCTCGCTTTTCTTCTTTTCTCACTGCATACTAACAGAAAAAAAAGCTACCACTCTTCAAACCACAAAAAAAC

>PCR_2

AAGGGGCAAACATCGCTGCCGATGCGCCGTGTTCGGCTGTCGCGCAGGGGCGCCCGGTTCTTTTTGTCAAGACCGACCTGTCCGGTGCCCTGAATGAACTGCAGGACGAGGCAGCGCGGCTATCGTGGCTGGCCACGACGGGCGTTCCTTGCGCAGCTGTGCTCGACGTTGTCACTGAAGCGGGAAGGGACTGGCTGCTATTGGGCGAAGTGCCGGGGCAGGATCTCCTGTCATCTCACCTTGCTCCTGCCGAGAAAGTATCCATCATGGCTGATGCAATGCGGCGGCTGCATACGCTTGATCCGGCTACCTGCCCATTCGACCACCAAGCGAAACATCGCATCGAGCGAGCACGTACTCGGATGGAAGCCGGTCTTGTCGATCAGGATGATCTGGACGAAGAGCATCAGGGGCTCGCGCCAGCCGAACTGTTCGCCAGGCTCAAGGCGCGCATGCCCGACGGCGAGGATCTCGTCGTGACCCATGGCGATGCCTGCTTGCCGAATATCATGGTGGAAAATGGCCGCTTTTCTGGATTCATCGACTGTGGCCGGCTGGGTGTGGCGGACCGCTATCAGGACATAGCGTTGGCTACCCGTGATATTGCTGAAGAGCTTGGCGGCGAATGGGCTGACCGCTTCCTCGTGCTTTACGGTATCGCCGCTCCCGATTCGCAGCGCATCGCCTTCTATCGCCTTCTTGACGAGTTCTTCTAGGTGCGATGTACACACCGTCTCGGAAGAGAACACTTGTGCGTTAGAGCGCTTGGCTGATGTGTTCGCATTCTTGGGTCGGCGCGCTGTGTTTCTGTGCCGATGCACTTGCCGCGTGCTGCTATGCCATTCCTCTCCTCGCTTTTCTTCTTTTCTCACTGCATACTAACAGAAAAGAAAAGCTACCACTCTTCAAACCACAAAAGAACAAGCAAGTCCCGCCCACTTCGGGTTGGTGACCCCTGACCTTGCGGTTCGTGAACATTGATACCAGAAGCACGGGAGTGTTCCTTCTCC

>PCR_3

CCACCTGAACCTGCGCAAATCTTGCGGTCTCGCATTATTGGATACCTTGGCGAAACGCCAAGCTAATACATGAACCAATCGGACGTGCTCCATTCACGCGGCGTTCGGGCTTGCCCGGGCGACGTAGCGAGTCGTCCAGCGAATGAATGAAAGTAAAACCAATGCCGCGGACGCGGCAGCAGCACTCAGAAGTGTTGACTCAATTCATTCCGTGCGAAAGCTGGTCTAACCAGCGTATTTTGACGAACAACTGCCCTATCAGCCAGTGATGGCCGTGTAGTGGACTGCCATGGCGTTGACGGGAGCGGGGGATTAGGGTTCGATTCCGGAGAGGGAGCCTGAGAAATAGCTACCACTTCTACGGAGGGCAGCAGGCGCGCAAATTGCCCAATGTCGATAAAAACGATGAGGCAGCGAAAAGAAATAGGCCTCCCAGCCACTCTGTGGTTGGGAGATACAATGGGGGATATTTAACGCCATCCAAAATCGAGTAACAATTGGAGGACAAGTCTGGTGCCAGCACCCGCGGTAATTCCAGCTCCAAAAGCGTATATTAACGCTGTTGCTGTTAGTGCGATGTAC

>PCR_3

CCACCTGCAATCGCCCAAATCTTGCGGTCTCGCATTATTGGATACCTTGGCGAAACGCCAAGCTAATACATGAACCAATCGGACGTGCTCCATTCACGCGGCGTTCGGGCTTGCCCGGGCGACGTAGCGAGTCGTCCAGCGAATGAATGAAAGTAAAACCAATGCCGCGGACGCGGCAGCAGCACTCAGAAGTGTTGACTCAATTCATTCCGTGCGAAAGCTGGTCTAACCAGCGTATTTTGACGAACAACTGCCCTATCAGCCAGTGATGGCCGTGTAGTGGACTGCCATGGCGTTGACGGGAGCGGGGGATTAGGGTTCGATTCCGGAGAGGGAGCCTGAGAAATAGCTACCACTTCTACGGAGGGCAGCAGGCGCGCAAATTGCCCAATGTCGATAAAAACGATGAGGCAGCGAAAAGAAATAGGCCTCCCAGCCACTCTGTGGTTGGGAGATACAATGGGGGATATTTAACGCCATCCAAAATCGAGTAACAATTGGAGGACAAGTCTGGTGCCAGCACCCGCGGTAATTCCAGCTCCAAAAGCGTATATTAACGCTGTTGCTGTTAGTGCGATGTACACACCGC

>PCR_4

GGGTCGGCGCGCTGTGTTTCTGTGCCGATGCACTTGCCGCGTGCTGCTATGCCATTCCTCTCCTCGCTTTTCTTCTTTTCTCACTGCATACTAACAGAAAAGAAAAGCTACCACTCTTCAAACCACAAAAGAACAAGCAAGTCCCGCCCACTTCGGGTTGGTGACCCTTGACCTTGCGGTTCGTGAACATTGATACAAGAAGCACGGGAGTGGTTCCTTTCCGATTCACGCATGTCATGCATGCCGGGGGGCGCCCGTGATTTTTACTGTGACTAAAGAAGCGCGACCAAAGCAGTCATTCGACTTGAATTAGAAAGCATGGGATAACAAAGGAGCAACCTACGGTACTACCGTTTTCGGCTTTTGTTGGTTTTAGAAGTCTGTGGGAGATTATGGTTTCGTGCGACCGGCGGCTGGGGTCCGTCCCCGCGCTTTTTGCTTTGTGGGGTTCGCCTTGCATTGTAGACTCGCGAGACCAGGAATGAAGGAGGGTTGTTCGGGGGAAAACGTACTGGGGCGTGAGAGGTGAAATTCTTAGACCGCCCCAAGACGAACTACAGCGAAGGCATTTTCCAAGGATACCTTCCTCAATCAAGAACCAAAGTGTGGAGATCGAAGATGATTAGAGACCATTGTAGTCCACACCACAAACGGTGACACCCATGAATTGGGGAATACT

>PCR_4

CTGGGTCGGCGCGCTGTGTTTCTGTGCCGATGCACTTGCCGCGTGCTGCTATGCCATTCCTCTCCTCGCTTTTCTTCTTTTCTCACTGCATACTAACAGAAAAGAAAAGCTACCACTCTTCAAACCACAAAAGAACAAGCGAGTCCCGCCCACTTCGGGTTGGTGACCCTTGACCTTGCGGTTCGTGAACATTGATACAAGAAGCACGGGAGTGGTTCCTTTCCGATTCACGCATGTCATGCATGCCGGGGGGCGCCCGTGATTTTTACTGTGACTAAAGAAGCGCGACCAAAGCAGTCATTCGACTTGAATTAGAAAGCATGGGATAACAAAGGAGCAACCTACGGTACTACCGTTTTCGGCTTTTGTTGGTTTTAGAAGTCTGTGGGAGATTATGGTTTCGTGCGACCGGCGGCTGGGGTCCGTCCCCGCGCTTTTTGCTTTGTGGGGTTCGCCTTGCATTGTAGACTCGCGAGACCAGGAATGAAGGAGGGTTGTTCGGGGGAAAACGTACTGGGGCGTGAGAGGTGAAATTCTTAGACCGCCCCAAGACGAACTACAGCGAAGGCATTTTCCAAGGATACCTTCCTCAATCAAGAACCAAAGTGTGGAGATCGAAGATGATTAGAGACCATTGTAGTCCACACCACAAANCGGTACACCCTAANTGAA

>PCR_4

TTTGGTCGGCGCGCTGTGTTTCTGTGCCGATGCACTTGCCGCGTGCTGCTATGCCATTCCTCTCCTCGCTTTTCTTCTTTTCTCACTGCATACTAACAGAAAAGAAAAGCTACCACTCTTCAAACCACAAAAGAACAAGCAAGTCCCGCCCACTTCGGGTTGGTGACCCTTGACCTTGCGGTTCGTGAACATTGATACAAGAAGCACGGGAGTGGTTCCTTTCCGATTCACGCATGTCATGCATGCCGGGGGGCGCCCGTGATTTTTACTGTGACTAAAGAAGCGCGACCAAAGCAGTCATTCGACTTGAATTAGAAAGCATGGGATAACAAAGGAGCAACCTACGGTACTACCGTTTTCGGCTTTTGTTGGTTTTAGAAGTCTGTGGGAGATTATGGTTTCGTGCGACCGGCGGCTGGGGTCCGTCCCCGCGCTTTTTGCTTTGTGGGGTTCGCCTTGCATTGTAGACTCGCGAGACCAGGAATGAAGGAGGGTTGTTCGGGGGAAAACGTACTGGGGCGTGAGAGGTGAAATTCTTAGACCGCCCCAAGACGAACTACAGCGAAGGCATTTTCCAAGGATACCTTCCTCAATCAAGAACCAAAGTGTGGAGATCGAAGATGATTAGAGACCATTGTAGTCCACACCACAAANCGGTACACCCNANTGNA

>PCR_4

ATGCCATTCTCTCCTCGCTTTTCTTCTTTTCTCACTGCATACTAACAGAAAAGAAAAGCTACCACTCTTCAAACCACAAAAGAACAAGCAAGTCCCGCCCACTTCGGGTTGGTGACCCTTGACCTTGCGGTTCGTGAACATTGATACAAGAAGCACGGGAGTGGTTCCTTTCCGATTCACGCATGTCATGCATGCCGGGGGGCGCCCGTGATTTTTACTGTGACTAAAGAAGCGCGACCAAAGCAGTCATTCGACTTGAATTAGAAAGCATGGGATAACAAAGGAGCAACCTACGGTACTACCGTTTTCGGCTTTTGTTGGTTTTAGAAGTCTGTGGGAGATTATGGTTTCGTGCGACCGGCGGCTGGGGTCCGTCCCCGCGCTTTTTGCTTTGTGGGGTTCGCCTTGCATTGTAGACTCGCGAGACCAGGAATGAAGGAGGGTTGTTCGGGGGAAAACGTACTGGGGCGTGAGAGGTGAAATTCTTAGACCGCCCCAAGACGAACTACAGCGAAGGCATTTTCCAAGGATACCTTCCTCAATCAAGAACCAAAGTGTGGAGATCGAAGATGATTAGAGACCATTGTAGTCCACACCACAAACGGTGACACCCATGAATTGGGGAATACT
