# Supplementary material for: CRISPR/Cas9‐induced disruption of Bodo saltans paraflagellar rod‐2 gene reveals its importance for cell survival
Source: Environ Microbiol. 2022 Feb 2;24(7):3051–62. doi: 10.1111/1462-2920.15918 (PMC9544060; doi:10.1111/1462-2920.15918)
Supplement: Supplementary file 5 — Data S4. Sequences highlighted in yellow confirm the eGFP‐Neo‐PFR plasmid integration [file EMI-24-3051-s005.docx]

Supplementary Data 4:

Sequences highlighted in yellow confirm the eGFP-Neo-PFR plasmid integration

>CYKH01000743.1 Bodo saltans genome assembly BSAL, scaffold BS_scaffold1667, whole genome shotgun sequence

CGAATGCTGGCGTAATTTCTGTGTTGAACAACGGTTGCCTACTTGCGCTCGTGCAAATGACTTTAGTGACGCGTTAAATTCACTGTTTTCAGCATTGCATCGCTGCGGCGTGAATACAAAAGCAGCTCGCGAAGCCTTGTTGCTCTCGTTTGAACAGCGTCATCATCAGTGGCTTGACGACCAATTGAAGGGAGATACGCTTGGCAGCAGCACCGCCAGAATCATGCGTGGATCAGCATTGATCAAACGTTCAACAAATAGCATCGAGCGTCGCATGTTTGATAGGGATGTGACCATCCGTGATTACCTTCCAGCTGGTGTGACTTGTATCCGACCCACAACGACGCTTGTGTTGCTAAGGCAGATGCAATACGCCCAGAAAAGGCCACACCAAGCGCTAAAGCATTGTGTTGGCATGAAACGAGTGAGTGATGCGATGCGCAGCATTTCGGCGATTCAAGATCGATCTTCGAACGCCGTTGGCCGTGGGACAGGTACTGAAAGCCAACGGGAACATGGACTCGAAGTATACTTTGATATGGACGAGAGTATTGGCGCTGAGGCTTGCGCTATTGCCTCTCGACTTGGGCATGAGAAGGTGTTGCATGTCTTCTGGAATGCTCTACGAAAGCGGGGGTGCATTTCTGCTGCCAAGTACTGCCTTGCTACGAGCTCCACTGCAACGGGCACTGACATTGTGAACGCCGTGCTCAATATCCCAACCAGCAATACCTCTTCAGCACCACCGCGACGCGTGGGTCTCAACTCACAAACCGTAAAAGTAATGCTTTTGGGAATGCATGGCGGGGTTGACAAACAGAATATCAGCGCAACAGTTTCTCGAGTGATACTACGGCAAATTCTGGAGGCAATTGGAAGGAATCCGGAATTGCTTGACGCGGACGTGTCATTTCTTTTGTTTCGGTGCCTTGGAGCTAGCGATGCAACGTTGGGTGCGAAGCTGCTGATGAACCAGTGGGATAGCCCCAATTCTGCTCGACCCTCTACGAACCACACACTCTCTCGCAGCGGTAAGACAGCAGCGACGCACGGACTCGATGCGATCAAACTCTCGCTTCGACGAGCTTCTGGAGACGAGAGCGTTGCCTCAGAAACTGATGACGCAAGCGAGGCTGAAGCAGCAGAGCGTGCAGTGCATTTTACCTCAATAATGCGCTCGCTTGGCAAATATCTCCACTCGAACCAAGTTGCTGCAAGCAATCGTACCCTACTTGCAGGTTCGTGTGGCTTTATCGCTGGTCTCACAACGTTGGAGTGCCGTAAATGGATTGCTGCTCTCGGACACGATTCACCATCTCGCTTCTTCTCAAACCTACTTGAAGAGCAAGCAAGTACATCTGAGGCGCCATCCAACTCTCCCCATTGTGTTGCAACAGCCCTTCTCAAGCATGTGTCAATTGACGATTTAAGAAGTCTTGTTGACCGTCAGCTTACTGCGATGGAGTCTGGCAAATTGACCCCTAACTCCATTCGCTCAGAAACCCTGCTAAGCCCAACAGCAGTGTTATTGGAGTTTGTTTCCCAAGTTTCAACGTCGACACGATTTGCTGACGTTTTGCGCGCATTGCGCTGAATCACATACTTTCCTCTATTTGATTCATATCAACGTTGCAGCGTTCACCAACAATATGACTGTTCAACAAATTTTTTTTCCTCCATCTCCCTATTCTTTTTTGAATGTAAAGCCCTTAGAGTACCATAACATGGCCGACCAACAACCCGAAGTTGCCGACATCACGCTGGAGGCGGCGCGCAAGCAGAAGATCCACAATCTGAAGCTGAAGACGTCGTGCCTGTCGAATGAGGAGTTCATCCAGGACCTGCACGTGTCCGACTGGTCCGAGACTCAGAAGCAGAAGCTGCAGGCCGCGCACGAGAAGGCGTCGGAGCTGCTTGCATCCGTGGAGAGCGGGACGAAGTGGAACCTGACCGCTGCGTACGACATCCAGAAGCTGATGCGCGTGTGCGGGCTGGAGATGTCTGTGCGCGAGCTGTACAAGCCGGAGGACAAGCCCCAGTTCATGGAGATTGTTGGCCTGAAGAAGACGCTGAACGAGCTGAAGCAGCACCGCAACAAGACCCGCATTGTGTCGTTCACCGGCACGATCGACAACGGCCTGACGAAGCTGGAGAAGGTCGAGGACGAGCTCCGCCGCTCGCAGCTCGACGCCACCGAGCTCGCGCAGGTGCCCGTTGCCGTGCTGAAGAACCTCGAGGAGTGCATGAACGTGACCGTCATCCAGTCCGCGCTGATGGGCAACGAGGAGCAGGTGAAGGCGCAGCTCGCTGCGATCGAGAAGGCGAAGGAGATCCGCAACGTAGCCGTCGCCGACGGTGAGATGGCGATTGCCGAGGAGCAGTTCTACATCAAGGCGCAGCTGCTGGAGCACCTTGTCGAGCTCGTTGCTGACAAGTTCCGCATCATTGGCCAGACTGACGACGAGAACAAGATGTTTGCGAAGATCCACGAGGTGCAGAAGAAGGCGTTCCAGGAGACGTCTGCGATGAAGGACGCGAAGCGCCGCCTGAAGCAGCGCTGCGAGGAGGACCTGAAGCACCTGCACGACGCCATCCAGAAGGCCGACCTTGAGGACGCCGAGGCGGTGAAGCGCTACATGGGCCAGAAGGACAAGTCCGAGAAGTTCGTGCGCGAGAACCTTGAGAAGCAGGACGAGTCGTGGCGCAAGATCCAGGAGCAGGAGCGCACGCTGCAGAAGCTCGGCACTGAGCGCTTCGAGGAGGTGAAGCGCCGCATCGAGGAGAACGACCGCGAGGAGAAGCGCAAGGTCGAGTACCAGCAGTTCCTCGACGTTGTTGGCCAGCACAAGAAGCTTCTTGAGCTGTCCGTGTACAACTGCGACTTGGCGATCCGTAGCATTGGCATCATCGAGGAGCTTGTTGCGGAGGGCTGCAGCGCAATCAAGGCCCGCTACGACAAGACGAACCAGGAGCTCGCTGACATGCGCCTGCAGGTCCACCAGGAGTACCTCGAGGCGTTCCGCCGCCTGTACAAGACGCTGGGCCAGCTTGTGTACAAGAAGGAGAAGCGCCTTGAGGAGATCGACCGCAACATCCGCACGACCCACATCCAGCTCGAGTTTGCCATCGAGACGTTCGACCCCAACGCGAAGAAGCACTCCGATAGCAAGAAGGAGCTGTACAAGCTGCGCGCGCAGGTGGAGGAGGAGCTCGAGATGCTCAAGGACAAGATGGCACAGTCGCTGGAGATGTTCGGCCCCACCGAGGATGCGCTGCACCAGGCCGGCATTGAGTTCGTGCACCCCGCTGAGGAGCTGGAGGAGAACAACGTTAGCCGCCGCAGCAAGATCGTCGAGTACCGTGCGCACCTTGCGAAGCAGGAGGAGGTGAAGATTGCGGCCGAGCGCGAGGAGCTCAAGCGCGCCAAGACGATCCAATCCCAGCAATACCGCGGCAAGCCCGTGCAGCAGCAGATCACCCAGTAAGTGTTTCGATGCTTGTAGAAGAGTGCGGAAGTCTGTATGTTGCTCGTCGGAAAGACGTGTGGACTTGTGTGATGAATGTTGAATGTACCTACGGTTAGCATTTTTTTGTGAACATTGTGTGACCAAAGTGTTTTATGATAGTAGACTTGGATGTGTGCTTGTTATTTTGGTGGACCTTGAGCTTTTTTTTTCCTTAGTAGAAAGGAGAGTCGCGCGATATCAGCGTCCTTTTCTCCTTTTTTTTGGTGAATGAATCAACAGACTCTGTCTGAGGCTCGGAGAATCCTTTCTGTGGTTGATGATCTTATTGTAGATTTGAATGTTGTGAGCCACTTGCCTTCGTACATGAGTGCGATGCCGCCCCAAGACTTGCAGCACATTACCAATGCGTTCGGAGGCGGCCAGAATGGCCGTGAAGTTCAGACTCAACTGAATGAGCACTTTGACCTGGAGAGGAAATTAGAATCTGCTGGTGGCGGTGAGGTTGCCGCGGAAGATGTTGCCGACCACCACTTGTCATGCCGCGCTCTGCTGGACACCCTTCGTGCTGCTGGCTACGGCCAAACATACCAACCCGCGTTCCCAGGAAGTGAGGGGATTCGAAACTTTTCGTACATCATGGGAGTTCTTCGAAGCTTGCTACATGACCGCTGCCACACCTCTGTTGAAGACGATGTGATCAAGTACACCATTCTACACGATACTGTCAACCGTGAGAAATCGGCGTCTGCTGATGTGCAGGCACTTAATCGCGAGTACCACAATGAGAAGGAGTCCCGGCGCATTGAGGTGGAGAAGCGGCAACAGGCCATCCGGAAAGTGCGCGAGGAGATCGAGCAGCTTCGTCAAGCGTCCGACACTGAGATGTCAAATTTCTTGAAACTGAGCAAAGAACTTGCGACCACCAACGAAGAGCGCTTTCAGCAAGAATTAGAGGAGTTGAAGACGAAGAAGGGTGAAATGTCCACTGAAACGGATCAGCTAGAATCGAAGTTCTTCAATGAAGAGAACGCACTGCGTGCTGCAAGATCCAAGAAGGAAACGACCATTAGCGCCACGATTAACGAATATGACACGCAACTTCAAAATCTCACGCAAACCATTTCAACCCTTCAGAAAGAGCTCGATGAGGATACAGAGCAGCTTGGGGAAGTAGAACGCGAGCTCCACCAGCTGAACCAGGATGCCAGTGAATACGAACTGGAGCGACGCATTGCTGAGCAACGCAAAGGCCACTACATGGACGTCAATGTTAGAATGGAGTCGCAGGCGAGGATTGTGCAGGCGTTCTTCCGTAGCTTTGCTGTTCGTTTGAAGGCTTCACAGAAGGGCAAGAAGAAGAGCAAGAAGAAGGATTAGACGCGGCGGGAAACCGATCGTGTGGTGTAGCTAGACATGCAAAAAAAAAAACAAAGTAGTTTGACGACTTCAATGTTTGTCAAAACAACGTGTACTTTACGAAAAAATGGCAGCACGATGTAAAAAAGGTCAACTCACTTTTGATTGCAACGCCTCAATTTCCTCGAGTTACCAAGCAACGATCAACGCATCGGTTCAGATGAGCACTATAACATATGAGCGTTTTGTTCTTTTTTTGAGTTTTTCCTACTTGGGAAAGTAAGAAAACGCAAAAATACAAACTTTCAACGTCGAAAAGAAATTAAGAAATGGCTCAGCAACCTCGCTACGCACAAACGCCCACCACTGGTGGCGCTCCAGCTGCTTTGGCCCCTTCGGCGTCATCATCAGCAGCAACGACGAGCACTAAGATCGGCGATCGCGCTTTTGATAAGATTGAGAAGACCTCAGCCGAGTTCTTTGCCATCACCTACGGCGCGCTAGTGCGACAAATGTTTATCGACCACAACGAGAGAGCCGACATTGTCAACACGCAGTTGGACCAGATGGGTGAACGCATTGGTGTGCGTTTGATTGAAGAGTATGCCGCACGAAGTGGTGCCCCTCCTTGCCGCTCGCCCGCACAGGCGGCGGACAGCGTGGCGAAGATTGGGCTGAAAATGTTTAATCCACAGAGGCCAAGCCAGTGGCTTCAACCGCCGCGATCTTCTGGTCCCGTATCATCGTCCACAGGTGCCGCAATCGGCGACGCAGCCAACGGCAGCAATGGAGGTGCCGTGTATTCCATATCGTTTGACGAGAACCCACTGAATGTGTTCGTGGAGCTGCCCGACGCACTTCGGCAGACACTCTGGTATAGCAATGTGCTATGCGGTGTGATTCGCGGAGGGTTGCAGCAAGTAGGCTTTGTCACATTGGTGTGGTACGTTCGCGACGTTCTTCGTGGGGATGATGTGAACGAGATTCGCATTCAGTTCCAAGGAAAAGAGCGCGAGACGTTCAAGGTTGACATGCAGAAGTAGTGCTGCTCCATCTCTTTCTCCAGATTGAGAGTGAGAGATTGAGAGAGGAGGCTGGGAGTTGAGAAGGTTGCTGTGCCAGAATTTGTTTTTTTTTTATATAGCGAGACCATGCGAAGAGAACGATCATAAAAATACAAATTGACAGAACATAAGTCTGCGTTCCTGCAATAAAAATGTGCTTACAAACAACTGTCGAGAAAGAGAGAGAGAGAGAGAGCGTACATAACACTAATCGATGGAGTACGCGCAAAAGTCTGCGCGGCTTCCCAAGACGCCTGAGGAGAAGGAAAGCTGGAAGCGAGTCATTATCATTTTGGAACACTGCCCCCTTTCTTCTGTCATGGGCGCCAAGGGCCATGAACTTCTGTCCGAAAAGCATAAAAACACGCACAGGAAACACAACCAAGACCCTGCTGAGTGGCGTCCTGACGTGGTACATCAGTGCTTACTCCATCTGCTAGACTCACCCCTAAGTCGGTCTGGACAACTTCAAATCTTCCTCCGAACAAAGAAGGGAGTTTGCATCGCTGTTGACCCACGTCTTCGAGTGCCTCGTTCCATGAGAATCTTTGAGAAAATGATGGTTAGCTGTTTGTACCGCATGAAAGTGCGTTCCACGTCTGGATACTTGAGCCTCATGAAGGTGGTGAAGAATCCCATCACAGATCACATTCCCGCGAATGTGCGACTGATTCGTGTTGAGAAGGACGGTGAGCTTGTGGACCCGTTTCTCCTCCCAAAGACGTTGGGCCGCTCCAACCATGAAGAAGCGGTGAAGCAGGTGGGGACAAGCACCAGCAGCAGTGGAGCTTTCGGTGCTCTTCACACGAAGAAAGCGGAGGAGACGTTTCGGCCGTTTGCGTTTGTGATTGGTGGTATGTCCAAGGGAGATGTGGATGCGGATTGGTGCCCGAAGCATCAAGTGCAGTCGATCCGGTTGGGTGATCGTAGTATGTCTGCGGCTGCGGTATGCTCAGCCATTGTTCATGGATTCGAAGAGACCTGGCTAGCTGAGGATAACAAACTCGCAAACCAATCATAAGAAAACGAAATAAATGCGAGCGGCGTCCCCGACGCGGTGTCGCTTTGTTACATCCCTCTTATCTACACCCGAGTAAACCAGCGCGCAATTATTAACGTTCGCGCGAGACATTCAATTCATCAAACAAAGGACTGCCACCATTGCTGCTTCCATTGCTGATTTTCCCACCTTTTTCTCTCGCAGTAGTAGATTAAAGTAAACGTGCGGGTCTGTCTTTTTTCCTTCTCTAGCATCTATGGGCTGCGCTGCATCGTCGTCCTCCGCAGCAGAGCGTCAGGAGTCTCCTGCTAAGAAGTACGCGAACAACAACAATCACAACGTTGCAGCCTCAACGAAGGTCGGCGGCGACAATGAACATCCCCTGGGTGGTGCCTCAAGTGCCGGTGGACGTCGCAGAACTCGGCGAGGTGATAGTAGCGCCAGCGGTGCAGCTGAAGATTATGTTGAGCTCTTCAAGGAAGAAAACATGAAGCCGCTCATGTGTCTCGACGAACGTATGAAAGTAGTTCAACTCTATGCCTCCACATTCCAGTCTGCACGGCAGGTCCTGGCGACAATTGGGCGCATCAAGACGACGATGAAGCGCTGGGTGCAGGCTGCAAGGCAACGACTGCAAGAGAAGAAAGCGGCACAGCAGAATTTTATGGTGGTCGTCAGCGGTACAGACGTCAGCAACGAGCACAATCCACTCTTTGACGCCAACGGCAATCACATCAACAACCGAGTCCCCGCGAGCCCAAAGGATGGTACAGGAAGCAGCAGTCATCAAGGAGGTGGTGGCGGACGGTTGCTCACCACAACGTCCGGCGTTGGAGGTGGTGCCGATCATCTCGTGGACGCTGACGGGTGGACCGTCGGAACTCCCGCGACAATGCGCCTTGGTGGATACAACGAGGACGACGAGGATGATACAGACGCCGCTGATTTACCGCTGTTCCCTGTCTTCCTGCAAGATTCGGACGGCGATGGTTTACTGTCGCCTGCCACCACCAGAGGAGCGGAGGACATCACTCCCGCTGTGACGCCACTGTTTGACCCAACACGTCGACAAAGTCGCCACGACGATGGTCAACAAAGTTGCAGCGAGACAAACACCAACACTGGTGACGTTGTTCCGCCACACCACTCAAAAGTACCCTCTGGGCGAGGGGAGCCCCGAAAAGCGCAGTTAGCCCTCGCCGGAGAAACACTCATTGCTCAGCGGTTGAAGCATCTAGGCCTTGAGCAGGTACTGATGGACAATAGCGACGGGAATTGTCAATTCCGATCATTGGCACATCAGATTCTTGGCGACGCTTCGCGACACGCAGAAGTTCGTAAAAAGATATGCGCTGCGATGATTGCCAAGCAGGAAGACGAGTACTCGTTCCTGTTTGAGTCG

>PFR gene_65378297

ACCAACAACCCGAAGTTGCCGACATCACGCTGGAGGCGGCGCGCAAGCAGAAGATCCACAATCTGAAGCTGAAGACGTCGTGCCTGTCGAATGAGGAGTTCATCCAGGACCTGCACGTGTCCGACTGGTCCGAGACTCAGAAGCAGAAGCTGCAGGCCGCGCACGAGAAGGCGTCGGAGCTGCTTGCATCCGTGGAGAGCGGGACGAAGTGGAACCTGACCGCTGCGTACGACATCCAGAAGCTGATGCGCGTGTGCGGGCTGGAGATGTCTGTGCGCGAGCTGTACAAGCCGGAGGACAAGCCCCAGTTCATGGAGATTGTTGGCCTGAAGAAGACGCTGAACGAGCTGAAGCAGCACCGCAACAAGACCCGCATTGTGTCGTTCACCGGCACGATCGACAACGGCCTGACGAAGCTGGAGAAGGTCGAGGACGAGCTCCGCCGCTCGCAGCTCGACGCCACCGAGCTCGCGCAGGTGCCCGTTGCCGTGCTGAAGAACCTCGAGGAGTGCATGAACGTGACCGTCATCCAGTCCGCGCTGATGGGCAACGAGGAGCAGGTGAAGGCGCAGCTCGCTGCGATCGAGAAGGCGAAGGAGATCCGCAACGTAGCCGTCGCCGACGGTGAGATGGCGATTGCCGAGGAGCAGTTCTACATCAAGGCGCAGCTGCTGGAGCACCTTGTCGAGCTCGTTGCTGACAAGTTCCGCATCATTGGCCAGACTGACGACGAGAACAAGATGTTTGCGAAGATCCACGAGGTGCAGAAGAAGGCGTTCCAGGAGACGTCTGCGATGAAGGACGCGAAGCGCCGCCTGAAGCAGCGCTGCGAGGAGGACCTGAAGCACCTGCACGACGCCATCCAGAAGGCCGACCTTGAGGACGCCGAGGCGGTGAAGCGCTACATGGGCCAGAAGGACAAGTCCGAGAAGTTCGTGCGCGAGAACCTTGAGAAGCAGGACGAGTCGTGGCGCAAGATCCAGGAGCAGGAGCGCACGCTGCAGAAGCTCGGCACTGAGCGCTTCGAGGAGGTGAAGCGCCGCATCGAGGAGAACGACCGCGAGGAGAAGCGCAAGGTCGAGTACCAGCAGTTCCTCGACGTTGTTGGCCAGCACAAGAAGCTTCTTGAGCTGTCCGTGTACAACTGCGACTTGGCGATCCGTAGCATTGGCATCATCGAGGAGCTTGTTGCGGAGGGCTGCAGCGCAATCAAGGCCCGCTACGACAAGACGAACCAGGAGCTCGCTGACATGCGCCTGCAGGTCCACCAGGAGTACCTCGAGGCGTTCCGCCGCCTGTACAAGACGCTGGGCCAGCTTGTGTACAAGAAGGAGAAGCGCCTTGAGGAGATCGACCGCAACATCCGCACGACCCACATCCAGCTCGAGTTTGCCATCGAGACGTTCGACCCCAACGCGAAGAAGCACTCCGATAGCAAGAAGGAGCTGTACAAGCTGCGCGCGCAGGTGGAGGAGGAGCTCGAGATGCTCAAGGACAAGATGGCACAGTCGCTGGAGATGTTCGGCCCCACCGAGGATGCGCTGCACCAGGCCGGCATTGAGTTCGTGCACCCCGCTGAGGAGCTGGAGGAGAACAACGTTAGCCGCCGCAGCAAGATCGTCGAGTACCGTGCGCACCTTGCGAAGCAGGAGGAGGTGAAGATTGCGGCCGAGCGCGAGGAGCTCAAGCGCGCCAAGACGATCCAATCCCAGCAATACCGCGGCAAGCCCGTGCAGCAGCAGATCACCCAGTAA

>PF1PR3_2KB BAND_WILD TYPEPFR2

NNANCANNNNNNNAGNNCNTNNNNNNNCATGNNNNACANCNTNCAGCANNNNCAATGANNNNTAGAGNNNGTGACNTCAGNTNCNNCGANGANNNNCAANTGNNNNAACTCATNNNNAGNAANCCTGNNAGCCNACAGCANTGTNNNAGTTNGTTTNCAAGNTTCAACGTCGANNACGANTTGCTGACGTTTGNGNNNATGCGCTGANTNNCATACTTTNCTNTATTTGATNANTTCACGGTGNNGGNGTCNNCNACNANNNNGNNNNTCCACCAANTTTTTTTNCNNCNTNTTCCTTNTTCTTTTTTGAATGTAAAGCCCTTAGAGTACCATAACATGGCTGACCAACAACCCGAAGTTGCCGACATCACGCTGGAGGCGGCGCGCAAGCAGAAGATCCACAATCTGAAGCTGAAGACGTCGTGCCTGTCGAATGAGGAGTTCATCCAGGACCTGCACGTGTCCGACTGGTCCGAGACTCAGAAGCAGAAGTTGCAGGCCGCGCACGAGAAGGCGTCGGAGCTGCTTGCATCCGTGGAGAGCGGGACGAAGTGGAACCTGACCGCTGCGTACGACATCCAGAAGCTGATGCGCGTGTGCGGGCTGGAGATGTCTGTGCGCGAGCTGTACAAGCCGGAGGACAAGCCCCAGTTCATGGAGATTGTTGGCCTGAAGAAGACGCTGAACGAGCTGAAGCAGCACCGCAACAAGACCCGCATTGTGTCGTTCACCGGCACGATCGACAACGGCCTGACGAAGCTGGAGAAGGTCGAGGACGAGCTCCGCCGCTCGCAGCTCGACGCCACCGAGCTCGCGCAGGTGCCCGTTGCCGTGCTGAAGAACCTCGAGGAGTGCATGAACGTGACCGTCATCCAGTCCGCGCTGATGGGCAACGAGGAGCAGGTGAAGGCGCAGCTCGCTGCGATCGAGAAGGCGAAGGAGATCCGCAACGTAGCNNTNNNNNANNNNNNNNNNNNNNNNNNNN

>PF1PR32KB BAND_WILD TYPEPFR2

NNNNNNNNNNNNNNNNNNNNNNTGNNNNNANGGGAACATGGACTCGAAGTATACTTTGATATGGACGAGAGTATTGGCGCTGAGGCTTGCGCTATTGCCTCTCGACTTGGGCATGAGAAGGTGTTGCATGTCTTCTGGAATGCTCTACGAAAGCGGGGGTGCATTTCTGCTGCCAAGTACTGCCTTGCTACGAGCTCCACTGCAACGGGCACTGACATTGTGAACGCCGTGCTCAATATCCCAACCAGCAATACCTCTTCAGCACCACCGCGACGCGTGGGTCTCAACTCACAAACCGTAAAAGTAATGCTTTTGGGAATGCATGGCGGGGTTGACAAACAGAATATCAGCGCAACAGTTTCTCGAGTGATACTACGGCAAATTCTGGAGGCAATTGGAAGGAATCCGGAATTGCTTGACGCGGACGTGTCATTTCTTTTGTTTCGGTGCCTTGGAGCTAGCGATGCAACGTTGGGTGCGAAGCTGCTGATGAACCAGTGGGATAGCCCCAATTCTGCTCGACCCTCTACGAACCACACACTCTCTCGCAGCGGTAAGACAGCAGCGACGCACGGACTCGATGCGATCAAACTCTCGCTTCGACGAGCTTCTGGAGACGAGAGCGTTGCCTCAGAAACTGATGACGCAAGCGAGGCTGAAGCAGCAGAGCGTGCAGTGCATTTTACCTCAATAATGCGCTCGCTTGGCAAATATCTCCACTCGAACCAAGTTGCTGCAAGCAATCGTACCCTACTTGCAGGTTCGTGTGGCTTTATCGCTGGTCTCACAACGTTGGAGTGCCGTAAATGGATTGCTGCTCTCGGACACNATTCACCATCTCGCTTCTTCTCAAACCTACTTGAAGAGCAAGCAAGTACATCTGAGGCGCCTTCCAACTCTCCCCATTGTGTTGCAACAGCCCTTCTCANGCATGTGTCAATTGACGATTTAANAANTCTTGTTGACCGTCAGCTTACTGCGATGGNANTCTGNNAATTGACCCCTAACTCNNTTCNCCTCNAAANNCCTGCTNANCCNCAACAGCAGTGTNNNTN

>eGFP Neo_Fused_PLASMID69 kDa paraflagellar rod protein fused EGFP/Neo_2512bp

TACCCTACTTGCAGGTTCGTGTGGCTTTATCGCTGGTCTCACAACGTTGGAGTGCCGTAAATGGATTGCTGCTCTCGGACACGATTCACCATCTCGCTTCTTCTCAAACCTACTTGAAGAGCAAGCAAGTACATCTGAGGCGCCATCCAACTCTCCCCATTGTGTTGCAACAGCCCTTCTCAAGCATGTGTCAATTGACGATTTAAGAAGTCTTGTTGACCGTCAGCTTACTGCGATGGAGTCTGGCAAATTGACCCCTAACTCCATTCGCTCAGAAACCCTGCTAAGCCCAACAGCAGTGTTATTGGAGTTTGTTTCCCAAGTTTCAACGTCGACACGATTTGCTGACGTTTTGCGCGCATTGCGCTGAATCACATACTTTCCTCTATTTGATTCATATCAACGTTGCAGCGTTCACCAACAATATGACTGTTCAACAAATTTTTTTTCCTCCATCTCCCTATTCTTTTTTGAATGTAAAGCCCTTAGAGTACCATAACATGGTGAGCAAGGGCGAGGAGCTGTTCACCGGGGTGGTGCCCATCCTGGTCGAGCTGGACGGCGACGTAAACGGCCACAAGTTCAGCGTGTCCGGCGAGGGCGAGGGCGATGCCACCTACGGCAAGCTGACCCTGAAGTTCATCTGCACCACCGGCAAGCTGCCCGTGCCCTGGCCCACCCTCGTGACCACCCTGACCTACGGCGTGCAGTGCTTCAGCCGCTACCCCGACCACATGAAGCAGCACGACTTCTTCAAGTCCGCCATGCCCGAAGGCTACGTCCAGGAGCGCACCATCTTCTTCAAGGACGACGGCAACTACAAGACCCGCGCCGAGGTGAAGTTCGAGGGCGACACCCTGGTGAACCGCATCGAGCTGAAGGGCATCGACTTCAAGGAGGACGGCAACATCCTGGGGCACAAGCTGGAGTACAACTACAACAGCCACAACGTCTATATCATGGCCGACAAGCAGAAGAACGGCATCAAGGTGAACTTCAAGATCCGCCACAACATCGAGGACGGCAGCGTGCAGCTCGCCGACCACTACCAGCAGAACACCCCCATCGGCGACGGCCCCGTGCTGCTGCCCGACAACCACTACCTGAGCACCCAGTCCGCCCTGAGCAAAGACCCCAACGAGAAGCGCGATCACATGGTCCTGCTGGAGTTCGTGACCGCCGCCGGGATCACTCTCGGCATGGACGAGCTGTACAAGATGCTTGAACAAGATGGATTGCACGCAGGTTCTCCGGCCGCTTGGGTGGAGAGGCTATTCGGCTATGACTGGGCACAACAGACAATCGGCTGCTCTGATGCCGCCGTGTTCCGGCTGTCAGCGCAGGGGCGCCCGGTTCTTTTTGTCAAGACCGACCTGTCCGGTGCCCTGAATGAACTGCAGGACGAGGCAGCGCGGCTATCGTGGCTGGCCACGACGGGCGTTCCTTGCGCAGCTGTGCTCGACGTTGTCACTGAAGCGGGAAGGGACTGGCTGCTATTGGGCGAAGTGCCGGGGCAGGATCTCCTGTCATCTCACCTTGCTCCTGCCGAGAAAGTATCCATCATGGCTGATGCAATGCGGCGGCTGCATACGCTTGATCCGGCTACCTGCCCATTCGACCACCAAGCGAAACATCGCATCGAGCGAGCACGTACTCGGATGGAAGCCGGTCTTGTCGATCAGGATGATCTGGACGAAGAGCATCAGGGGCTCGCGCCAGCCGAACTGTTCGCCAGGCTCAAGGCGCGCATGCCCGACGGCGAGGATCTCGTCGTGACCCATGGCGATGCCTGCTTGCCGAATATCATGGTGGAAAATGGCCGCTTTTCTGGATTCATCGACTGTGGCCGGCTGGGTGTGGCGGACCGCTATCAGGACATAGCGTTGGCTACCCGTGATATTGCTGAAGAGCTTGGCGGCGAATGGGCTGACCGCTTCCTCGTGCTTTACGGTATCGCCGCTCCCGATTCGCAGCGCATCGCCTTCTATCGCCTTCTTGACGAGTTCTTCTAGGTGTTTCGATGCTTGTAGAAGAGTGCGGAAGTCTGTATGTTGCTCGTCGGAAAGACGTGTGGACTTGTGTGATGAATGTTGAATGTACCTACGGTTAGCATTTTTTTGTGAACATTGTGTGACCAAAGTGTTTTATGATAGTAGACTTGGATGTGTGCTTGTTATTTTGGTGGACCTTGAGCTTTTTTTTTCCTTAGTAGAAAGGAGAGTCGCGCGATATCAGCGTCCTTTTCTCCTTTTTTTTGGTGAATGAATCAACAGACTCTGTCTGAGGCTCGGAGAATCCTTTCTGTGGTTGATGATCTTATTGTAGATTTGAATGTTGTGAGCCACTTGCCTTCGTACATGAGTGCGATGCCGCCCCAAGACTTGCAGCACATTACCAATGCGTTCGGAGGCGGCCAGAATGGCCGTGAAGTTCAGACTCAACTGAATGAGCACTTTGACCTGGAGAGGAAATTAGAATCTGCTGGTGGCGGTGAGGTTGCCGCGGAAGATGT

>PF2_PF4_1.5KB

TGTACAAGATGCTTGAACAAGATGGATTGCACGCAGGTTCTCCGGCCGCTTGGGTGGAGAGGCTATTCGGCTATGACTGGGCACAACAGACAATCGGCTGCTCTGACGCCGCCGTGTTCCGGCTGTCAGCGCAGGGGCGCCCGGTTCTTTTTGTCAAGACCGACCTGTCCGGTGCCCTGAATGAACTGCAGGACGAGGCAGCGCGGCTATCGTGGCTGGCCACGACGGGCGTTCCTTGCGCAGCTGTGCTCGACGTTGTCACTGAAGCGGGAAGGGACTGGCTGCTATTGGGCGAAGTGCCGGGGCAGGATCTCCTGTCATCTCACCTTGCTCCTGCCGAGAAAGTATCCATCATGGCTGATGCAATGCGGCGGCTGCATACGCTTGATCCGGCTACCTGCCCATTCGACCACCAAGCGAAACATCGCATCGAGCGAGCACGTACTCGGATGGAAGCCGGTCTTGTCGATCAGGATGATCTGGACGAAGAGCATCAGGGGCTCGCGCCAGCCGAACTGTTCGCCAGGCTCAAGGCGCGCATGCCCGACGGCGAGGATCTCGTCGTGACCCATGGCGATGCCTGCTTGCCGAATATCATGGTGGAAAATGGCCGCTTTTCTGGATTCATCGACTGTGGCCGGCTGGGTGTGGCGGACCGCTATCAGGACATAGCGTTGGCTACCCGTGATATTGCTGAAGAGCTTGGCGGCGAATGGGCTGACCGCTTCCTCGTGCTTTACGGTATCGCCGCTCCCGATTCGCAGCGCATCGCCTTCTATCGCCTTCTTGACGAGTTCTTCTAGGTGTTTCGATGCTTGTAGAAGAGTGCGGAAGTCTGTATGTAATCACTAGTGCGGCCGCCTGCAGGTCGACCATATGGGAGAGCTCCCAACGCGTTGGATGCATAGCTTGAGTATNCTATAGNNNNNNNNNNNNNNNNNNNNN

>PF2_PF4_1.5KB

NNNNNNNNNNNNNNGGCGATTGGGCCCGACGTCGCATGCTCCCGGCCGCCATGGCCGCGGGATTTGTAAAGCCCTTAGAGTACCATAACATGGTGAGCAAGGGCGAGGAGCTGTTCACCGGGGTGGTGCCCTTCCTGGTCGAGCTGGACGGCGACGTAAACGGCCACAGGTTCAGCGTGTCCGGCGAGGGCGAGGGCGATGCCACCTACGGCAAGCTGACCCTGAAGTTCATCTGCACCACCGGCAAGCTGCCCGTGCCCTGGCCCACCCTCGTGACCACCCTGACCTACGGCGTGCAGTGCTTCAGCCGCTACCCCGACCACATGAAGCAGCACGACTTCTTCAAGTCCGCCATGCCCGAAGGCTACGTCCAGGAGCGCACCATCTTCTTCAAGGACGACGGCAACTACAAGACCCGCGCCGAGGTGAAGTTCGAGGGCGACACCCTGGTGAACCGCATCGAGCTGAAGGGCATCGACTTCAAGGAGGACGGCAACATCCTGGGGCACAAGCTGGAGTACAACTACAACAGCCACAACGTCTATATCATGGCCGACAAGCAGAAGAACGGCATCAAGGTGAACTTCAAGATCCGCCACAACATCGAGGACGGCAGCGTACAGCTCGCCGACCACTACCAGCAGAACACCCCCATCGGCGACGGCCCCGTGCTGCTGCCCGACAACCACTACCTGAGCACCCAGTCCGCCCTGAGCAAAGACCCCAACGAGAAGCGCGATCACATGGTCCTGCTGGAGTTCGTGACCGCCGCCGGGATCACTCTCGGCATGGACGAGCTGTACAAGATGCTTGAACAAGATGGATTGCACGCAGGTTCTCCGGCCGCTTGGGTGGAGAGGCTATTCGGCTATGACTGGGCACAACAGACAATCGGCTGCTCTGACGCCGCCGTGNNN

>PF6PR5_INEGRATION CROSSOVER_PCR#2

NNNNNNNNNNNNNNNNNNNNNNNCGAGCTNNNCGGTAGNCGNCCNGCGTCCNCGATTCAGCAGCGCATCGCCTTCTATCGCCTTCTTGACGAGTTCTTCTGATGAGCTGTCCGTGTACAACTGCGACTTGGCGATCCGTAGCATTGGCATCATCGAGGAGCTTGTTGCGGAGGGCTGCAGCGCAATCAAGGCCCGCTACGACAAGACGAACCAGGAGCTCGCTGACATGCGCCTGCAGGTCCACCAGGAGTACCTCGAGGCGTTCCGCCGCCTGTACAAGACGCTGGGCCAGCTTGTGTACAAGAAGGAGAAGCGCCTTGAGGAGATCGACCGCAACATCCGCACGACCCACATCCAGCTCGAGTTTGCCATCGAGACGTTCGACCCCAACGCGAAGAAGCACTCCGATAGCAAGAAGGAGCTGTACAAGCTGCGCGCGCAGGTGGAGGAGGAGCTCGAGATGCTCAAGGACAAGATGGCACAGTCGCTGGAGATGTTCGGCCCCACCGAGGATGCGCTGCACCAGGCCGGCATTGAGTTCGTGCACCCCGCTGAGGAGCTGGAGGAGAACAACGTTAGCCGCCGCAGCAAGATCGTCGAGTACCGTGCGCACCTTGCGAAGCAGGAGGAGGTGAAGATTGCGGCCGAGCGCGAGGAGCTCAAGCGCGCCAAGACGATCCAATCCCAGCAATACCGCGGCAAGCCCGTGCAGCAGCAGATCACCCAGTAAGTGTTTCGATGCTTGTAGAAGAGTGCGGAAGTCTGTATGTTGCTCGTCGGAAAGACGTGTGGACTTGTGTGATGAATGTTGAATGTACCTACGGTTAGCATTTTTTTGTGAACATTGTGTGACCAAAGTGTTTTATGATAGTAGACTTGGATGTGTGCTTGTTATTTTGGTGGACCTTGAGCTTTTTTTTTCCTTAGTAGAAAGGANNAGTCNNNNCGANNNTCAGCGTCCTTTTNNNCTTTTTTTTGGNNAATGNATCAACAANNNNNNNGTNNNN

>PF6PR5_integration_CROSSOVER_PCR#2

NAAGNANNNNTCCNANNANNNNNNAGNAGCTNNTACAAGCTGCGNNNNCAGNTGGAGNAGGAGCTNGAGATGCTCAAGGANAGATGGCACAGTCGCTGNAGATGTTCGNCCCCACCGAGGATGCGCTGCACCAGGCCGGCATTGAGTTCGTGCACCCCGCTGAGGAGCTGGAGGAGAACAACGTTAGCCGCCGCAGCAAGATCGTCGAGTACCGTGCGCACCTTGCGAAGCAGGAGGAGGTGAAGATTGCGGCCGAGCGCGAGGAGCTCAAGCGCGCCAAGACGATCCAATCCCAGCAATACCGCGGCAAGCCCGTGCAGCAGCAGATCACCCAGTAAGTGTTTCGATGCTTGTAGAAGAGTGCGGAAGTCTGTATGTTGCTCGTCGGAAAGACGTGTGGACTTGTGTGATGAATGTTGAATGTACCTACGGTTAGCATTTTTTTGTGAACATTGTGTGACCAAAGTGTTTTATGATAGTAGACTTGGATGTGTGCTTGTTATTTTGGTGGACCTTGAGCTTTTTTTTTCCTTAGTAGAAAGGAGAGTCGCGCGATATCAGCGTCCTTTTCTCCTTTTTTTTGGTGAATGAATCAACAGACTCTGTCTGAGGCTCGGAGAATCCTTTCTGTGGTTGATGATCTTATTGTAGATTTGAATGTTGTGAGCCACTTGCCTTCGTACATGAGTGCGATGCCGCCCCAAGACTTGCAGCACATTACCAATGCGTTCGGAGGCGGCCAGAATGGCCGTGAAGTTCAGACTCAACTGAATGAGCACTTTGACCTGGAGAGGAAATTAGAATCTGCTGGTGGCGGTGAGGTTGCCGCGGAAGATGTTGCCGACCACCACTTGTCATGCCGCGCTCTGCTGGACACCCTTCGTGCTGCTGGCTACGGCCAAACATACCAACCCGCGTTCCCAGGAAGTGAGGGGATTNGAAACTTTTCGTACATCATGGGAGTTGNTTNGAACNT

>PF1PR3_lower band_KO PFR_1.5 KB_NHEJ

CACTGACATTGTGAACGCCGTGCTCAATATCCCAACCAGCAATNCCTNTTCAGCACCACCGCGACGCGTGGGTCTCAACTCACAAACCGTAAAAGTAATGCTTTTGGGAATGCATGGCGGGGTTGACAAACAGAATATCAGCGCAACAGTTTCTCGAGTGATACTACGGCAAATTCTGGAGGCAATTGGAAGGAATCCGGAATTGCTTGACGCGGACGTGTCATTTCTTTTGTTTCGGTGCCTTGGAGCTAGCGATGCAACGTTGGGTGCGAAGCTGCTGATGAACCAGTGGGATAGCCCCAATTCTGCTCGACCCTCTACGAACCACACACTCTCTCGCAGCGGTAAGACAGCAGCGACGCACGGACTCGATGCGATCAAACTCTCGCTTCGACGAGCTTCTGGAGACGAGAGCGTTGCCTCAGAAACTGATGACGCAAGCGAGGCTGAAGCAGCAGAGCGTGCAGTGCATTTTACCTCAATAATGCGCTCGCTTGGCAAATATCTCCACTCGAACCAAGCTGCTGCAAGCAATCGTACCCTACTTGCAGGTTCGTGTGGCTTTATCGCTGGTCTCACAACGTTGGAGTGCCGTAAATGGATTGCTGCTCTCGGACACGATTCACCATCTCGCTTCTTCTCAAACCTACTTGAAGAGCAAGCAAGTACATCTGAGGCGCCATCCAACTCTCCCCATTGTGTTGCAACAGCCCTTCTCAAGCATGTGTCAATTGACGATTTAAGAAGTCTTGTTGACCGTCAGCTTACTGCGATGGAGTCTGGCAAATTGACCCCTAACTCCATTCGCTCAGAAACCCTGCTAAGCCCAACAGCAGTGTTATTGGAGTTTGTTTCCCAAGTTTCAACGTCGACACGATTTGCTGACGTTTTGCGCGCATTGCGCTGAATCACATACTTTCCTCTATTTGATTCATATCAACGTTGCAGCGTTCACAATCCCGCGGCCATGGCGGCCGGGAGCATGCGACGTCGGGCCCAATCGCC

>PF1PR3_LOWERBAND_KO PFR_1.5 KB_NHEJ

TGANCTATAGAATACTCAAGCTATGCATCCAACGCGTTGGGAGCTCTCCCATATGGTCGACCTGCAGGCGGCCGCACTAGTGATTGATTCAAGATCGATCTTCGAACGCCGTTGGCCGTGGGACAGGTACTGAAAGCCAACGGGAACATGGACTCGAAGTATACTTTGATATGGACGAGAGTATTGGCGCTGAGGCTTGCGCTATTGCCTCTCGACTTGGGCATGAGAAGGTGTTGCATGTCTTCTGGAATGCTCTACGAAAGCGGGGGTGCATTTCTGCTGCCAAGTACTGCCTTGCTACGAGCTCCACTGCAACGGGCACTGACATTGTGAACGCCGTGCTCAATATCCCAACCAGCAATACCTCTTCAGCACCACCGCGACGCGTGGGTCTCAACTCACAAACCGTAAAAGTAATGCTTTTGGGAATGCATGGCGGGGTTGACAAACAGAATATCAGCGCAACAGTTTCTCGAGTGATACTACGGCAAATTCTGGAGGCAATTGGAAGGAATCCGGAATTGCTTGACGCGGACGTGTCATTTCTTTTGTTTCGGTGCCTTGGAGCTAGCGATGCAACGTTGGGTGCGAAGCTGCTGATGAACCAGTGGGATAGCCCCAATTCTGCTCGACCCTCTACGAACCACACACTCTCTCGCAGCGGTAAGACAGCAGCGACGCACGGACTCGATGCGATCAAACTCTCGCTTCGACGAGCTTCTGGAGACGAGAGCGTTGCCTCAGAAACTGATGACGCAAGCGAGGCTGAAGCAGCAGAGCGTGCAGTGCATTTTACCTCAATAATGCGCTCGCTTGGCAAATATCTCCACTCGAACCAAGCTGCTGCAAGCAATCGTACCCTACTTGCAGGTTCGTGTGGCTTTATCGCTGGTCTCACAACGTTGGAGTGCCGTAAATGGNTTGCTGCTCTCGGACACGATTCACCATCTCGCTTCTTCTCAAACCTACTTGAANAGCAAGCAAGTACATCTGAGNNGCCATCC
